# XRN2 Autoregulation and Control of Polycistronic Gene Expresssion in *Caenorhabditis elegans*

**DOI:** 10.1371/journal.pgen.1006313

**Published:** 2016-09-15

**Authors:** Takashi S. Miki, Sarah H. Carl, Michael B. Stadler, Helge Großhans

**Affiliations:** 1 Friedrich Miescher Institute for Biomedical Research, Basel, Switzerland; 2 Swiss Institute of Bioinformatics, Basel, Switzerland; University of Cambridge, UNITED KINGDOM

## Abstract

XRN2 is a conserved 5’→3’ exoribonuclease that complexes with proteins that contain XRN2-binding domains (XTBDs). In *Caenorhabditis elegans* (*C*. *elegans*), the XTBD-protein PAXT-1 stabilizes XRN2 to retain its activity. XRN2 activity is also promoted by 3'(2'),5'-bisphosphate nucleotidase 1 (BPNT1) through hydrolysis of an endogenous XRN inhibitor 3’-phosphoadenosine-5'-phosphate (PAP). Here, we find through unbiased screening that loss of *bpnt-1* function suppresses lethality caused by *paxt-1* deletion. This unexpected finding is explained by XRN2 autoregulation, which occurs through repression of a cryptic promoter activity and destabilization of the *xrn-2* transcript. De-repression appears to be triggered such that more robust XRN2 perturbation, by elimination of both PAXT-1 and BPNT1, is less detrimental to worm viability than absence of PAXT-1 alone. Indeed, we find that two distinct XRN2 repression mechanisms are alleviated at different thresholds of XRN2 inactivation. Like more than 15% of *C*. *elegans* genes, *xrn-2* occurs in an operon, and we identify additional operons under its control, consistent with a broader function of XRN2 in polycistronic gene regulation. Regulation occurs through intercistronic regions that link genes in an operon, but a part of the mechanisms may allow XRN2 to operate on monocistronic genes in organisms lacking operons.

## Author Summary

XRN2 is a conserved eukaryotic protein that controls gene expression by degrading or processing various types of RNA. Here we find that XRN2 negatively regulates its own levels in the nematode *C*. *elegans*. In response to reduction of XRN2 activity, this self-repression is alleviated, increasing *xrn-2* mRNA and thus protein production, which restores robust XRN2 activity. Although XRN2 and its upstream gene are transcribed from a single promoter as a gene expression unit called “operon”, XRN2 regulates only itself. It does so by inactivating a cryptic promoter that exists between the two genes and by destabilizing its own nascent transcript. Many other *C*. *elegans* genes (>15%) occur in operons, and we identify additional operons that XRN2 regulates through an analogous mechanism. Thus we find a novel function of XRN2 in modulating expression of genes in operons including itself. As one of the mechanisms could operate on genes outside operons, XRN2 may also regulate gene expression in organisms lacking operonic gene organization.

## Introduction

Polycistronic gene expression is common in prokaryotes: multiple genes are arranged tandemly and transcribed from a single promoter, as one RNA precursor. This organization of genes into an operon permits regulation of functionally related genes in one unit. By contrast, protein-coding genes in eukaryotes are usually organized monocistronically, i.e., one promoter drives the expression of one gene. However, operons do occur in some eukaryotes, such as the nematode *Caenorhabditis elegans* (*C*. *elegans*) [1], and the fly *Drosophila melanogaster* [2, 3, 4]. In fact, at least 15% of *C*. *elegans* genes are predicted to be in operons [5, 6].

Although the polycistronic transcript is the template for protein synthesis in prokaryotes, the individual cistrons in *C*. *elegans* are separated prior to translation, in the nucleus, by a process termed *trans*-splicing [7, 8]. This process is mechanistically similar to *cis*-splicing, which uses the spliceosome to excise the introns and fuse the exons of eukaryotic pre-mRNAs. However, rather than joining two fragments of the same precursor, it links a 5’-capped, 22-nucleotide RNA sequence, the splice-leader (SL), which is transcribed separately, to a splice acceptor site on the nascent transcript. *Trans*-splicing occurs on both monocistronic genes and genes in operons, but with splice-leaders of different sequences: whereas monocistronic genes and those most promoter-proximal in operons are spliced to SL1, the other operon-contained genes exhibit preferential albeit not exclusive splicing to SL2 [1, 5]. Moreover, for downstream genes in operons, *trans*-splicing occurs subsequent to cleavage of the immediately upstream pre-mRNA at the 3’ end [7, 8].

Unexpectedly, *C*. *elegans* operons do not appear to be enriched for functionally related genes [9] and, consistent with diversity in function, transcript levels of genes in an operon can vary [9]. One mechanism that can uncouple genes within an operon is the existence of an internal promoter that permits expression of downstream genes independently of the first gene. Indeed, more than a quarter of *C*. *elegans* operons are estimated to have internal promoters [10] in intercistronic regions (ICRs), i.e., the intergenic space between neighboring genes, typically reflected by an unusually large ICR length of ≥ 500 base pairs [5]. Varying activities of the operon promoter and an internal promoter can thus generate quantitative and spatial diversity in expression of genes in shared operons [10, 11]. Additional mechanisms may further diversify expression patterns, across tissues, development, or in response to environmental cues, but are less well understood.

XRN2 is a 5’→3’ exoribonuclease that is conserved in eukaryotes. XRN2 is predominantly localized in the nucleus, and several nuclear RNA species have been reported as its targets [12, 13]. XRN2 recognizes RNA with a 5’ monophosphate and degrades it to mononucleotides [14, 15]. In yeast, the activity of the XRN2 orthologue Rat1p was found to be inhibited by 3’-phosphoadenosine-5'-phosphate (PAP), a byproduct of sulfate assimilation [16]. PAP is generated from 3'-phosphoadenosine-5'-phosphosulfate (PAPS) by sulfotransferase and converted to adenosine 5’-monophosphate (AMP) and phosphate (Pi) by 3'(2'),5'-bisphosphate nucleotidase (BPNT) [17]. As expected from BPNT’s function as a negative regulator of XRN2’s negative regulator PAP, loss of BPNT function has been shown to recapitulate or enhance loss-of-function phenotypes of XRN2 homologues in yeast [16] and plants [18, 19, 20, 21].

In *C*. *elegans*, XRN2 is essential for embryogenesis, larval development and fertility. The *xrn-2* gene is encoded in an operon as the second gene and shows ubiquitous expression throughout development [22]. We have recently reported that XRN2 is stabilized by forming a complex with PAXT-1 in *C*. *elegans* [23, 24]. *paxt-1 null* (*paxt-1(0)*) animals cannot survive at temperatures ≥ 26°C due to degradation of XRN2. This phenotype is suppressed by an increased *xrn-2* gene dosage [23] and recapitulated when a single amino acid change within PAXT-1 specifically prevents its binding to XRN2 [24], and thus a consequence of impaired XRN2 function.

Here we identify a loss-of-function mutation in the *ZK430*.*2*/*bpnt-1* gene as a suppressor of *paxt-1(0)* lethality. Depletion of BPNT1 protein induces accumulation of *xrn-2* mRNA and thus XRN2 protein through inhibition of XRN2 activity. This autoregulation requires the ICR upstream of *xrn-2* and does not affect the expression of its polycistronic partner *rpl-43*. A genome-wide RNA sequencing analysis identifies a subset of operons that are controlled by XRN2 in an analogous manner, revealing a novel role of XRN2 in polycistronic gene expression.

## Results

### Loss of *bpnt-1* function rescues *paxt-1(0)* animals from larval arrest

**Fig 1:**
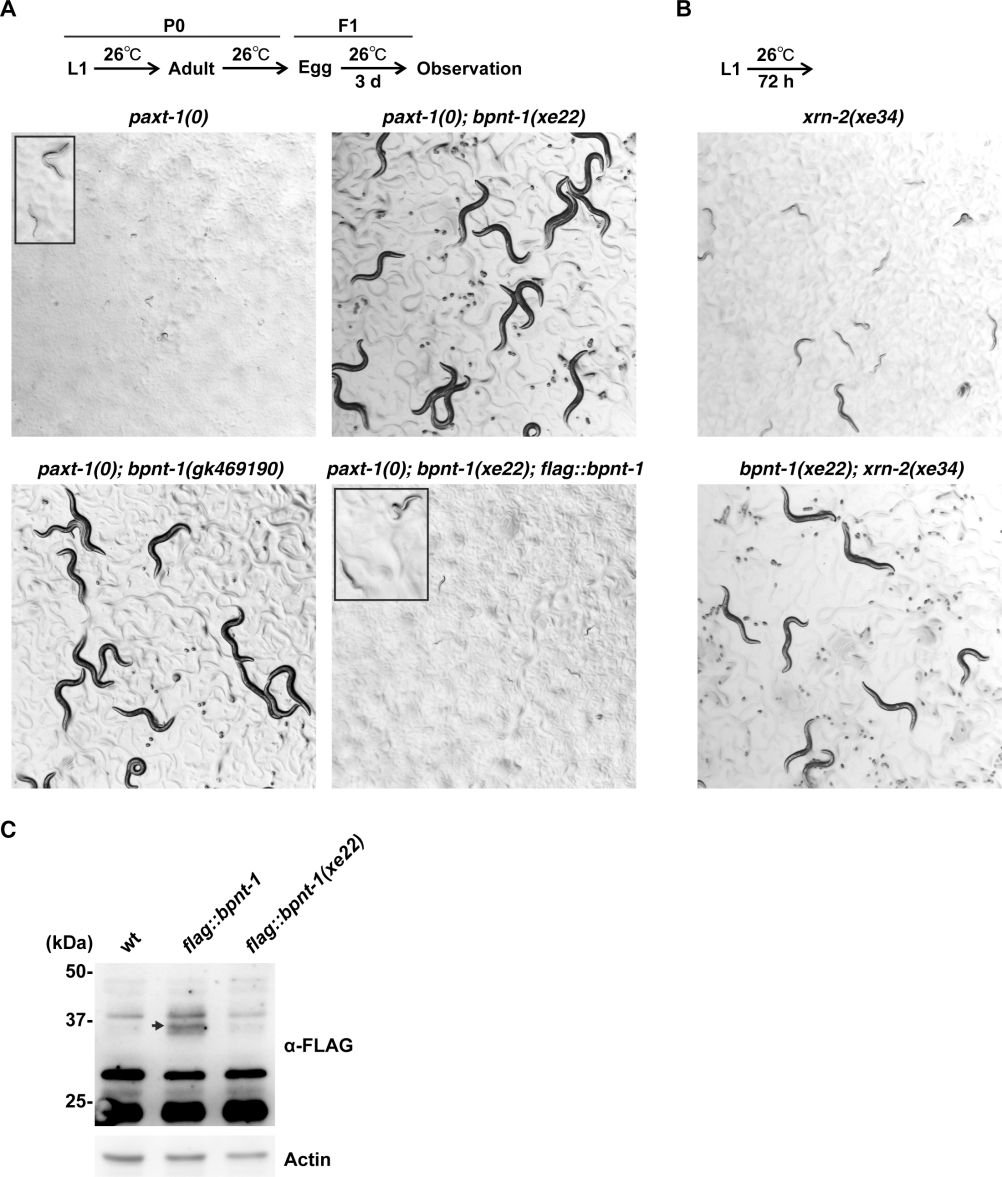
Loss of *bpnt-1* function rescues *paxt-1(0)* animals from larval arrest. (A) Animals of indicated genotypes were cultured at 26°C from L1 until egg laying. After 3 days, hatched progeny were observed by stereomicroscopy at the same magnification. Insets show arrested larvae observed at higher magnification. (B) Animals of indicated genotypes were cultured at 26°C from L1 for 72 hours and observed. (C) Wild-type (wt) N2, *flag*::*bpnt-1* or *flag*::*bpnt-1(xe22)* animals were cultured from L1 to L4 at 26°C for 30 hours and harvested. FLAG::BPNT1 and Actin were detected by western blot using anti-FLAG and anti-Actin antibodies, respectively. The arrow indicates FLAG::BPNT1.

We have recently reported that XRN2 is stabilized by forming a complex with PAXT-1 and that PAXT-1 is required for larval development of *C*. *elegans* animals at temperatures ≥ 26°C [23, 24]. In order to gain more insight into regulation of XRN2 stability or expression, we performed an ethyl methanesulfonate (EMS) mutagenesis screen for mutant animals that could survive such an elevated temperature in the absence of PAXT-1. A genomic DNA sequencing analysis of an isolated mutant identified a nonsense mutation in the *ZK430*.*2* gene. As the gene encodes a protein that shows structural and functional conservation with the human BPNT1 ([25] and see below), we named it *bpnt-1*. For simplicity, we will refer to the *C*. *elegans* protein as BPNT1. The mutant allele, *bpnt-1(xe22)*, completely suppressed the larval arrest phenotype of *paxt-1(0)* (Fig 1A). Since the larval arrest phenotype of *paxt-1(0)* animals seems exclusively caused by destabilization of XRN2 [23, 24], we examined if the *bpnt-1(xe22)* allele could suppress *xrn-2* phenotypes. We utilized *xrn-2(xe34)*, a temperature-sensitive *xrn-2* allele that we obtained from a genetic screen that will be described elsewhere. The *xrn-2(xe34)* allele has a missense mutation that changes a glutamic acid at position 699 to lysine, and the mutant animals show developmental defects and are not maintainable above 25°C (S1 Fig). *xrn-2(xe34)* animals were arrested as larvae when cultured at 26°C, while *bpnt-1(xe22); xrn-2(xe34)* animals developed to adult (Fig 1B). Thus, the *bpnt-1(xe22)* allele can suppress both the *paxt-1* and *xrn-2* phenotypes.

The *C*. *elegans* BPNT1 protein consists of 319 amino-acids, and its molecular weight is estimated to be 34.4 kDa. The mutation identified in the *bpnt-1(xe22)* allele changes tryptophan at position 294 to stop (W294*). A strain with the same mutation, VC40114, had been isolated in the Million Mutation Project [26]. In order to confirm that the *bpnt-1(xe22)* allele was responsible for suppression of the *paxt-1(0)* phenotype, we removed unrelated mutations from the VC40114 strain by outcrossing three times followed by crossing the *bpnt-1(gk469190)* allele into the *paxt-1(0)* background. The *bpnt-1(gk469190)* allele, like the *bpnt-1(xe22)* allele, completely suppressed the larval arrest phenotype of *paxt-1(0)* (Fig 1A).

Given that BPNT1 negatively regulates a negative regulator of XRN2 PAP in yeast [16], it seemed possible that *xe22* encoded a gain-of-function allele that enhanced BPNT1 activity on PAP. However, when modeling *C*. *elegans* BPNT1 on the published crystal structure of rat BPNT1 [26], we noticed that truncation of the C-terminal region in BPNT1(W294*) would result in exposure of the central β-sheet domain to solvent, and thus presumably destabilize the protein (S2 Fig). To test this experimentally, we expressed FLAG-tagged protein in wild-type animals from the *flag*::*bpnt-1* and the *flag*::*bpnt-1(xe22)* transgenes, respectively, which we integrated in the same genomic locus using Mos1-mediated single copy insertion (MosSCI) [27]. As predicted, the FLAG-tagged wild-type BPNT1 was detected at the expected molecular size (~35 kDa), while the mutant protein was essentially absent (Fig 1C). Hence, these data suggested that *xe22* results in a loss- rather than gain-of-function allele. We confirmed this by crossing the single copy-integrated *flag*::*bpnt-1* transgene into *paxt-1(0); bpnt-1(xe22)* animals, which reinstated temperature-sensitive lethality (Fig 1A). Thus, *xe22* is a loss-of-function allele that confers suppression of *paxt-1(0)* mutant lethality.

### BPNT1 depletion increases mRNA and protein levels of XRN2 without stimulating its promoter activity

**Fig 2:**
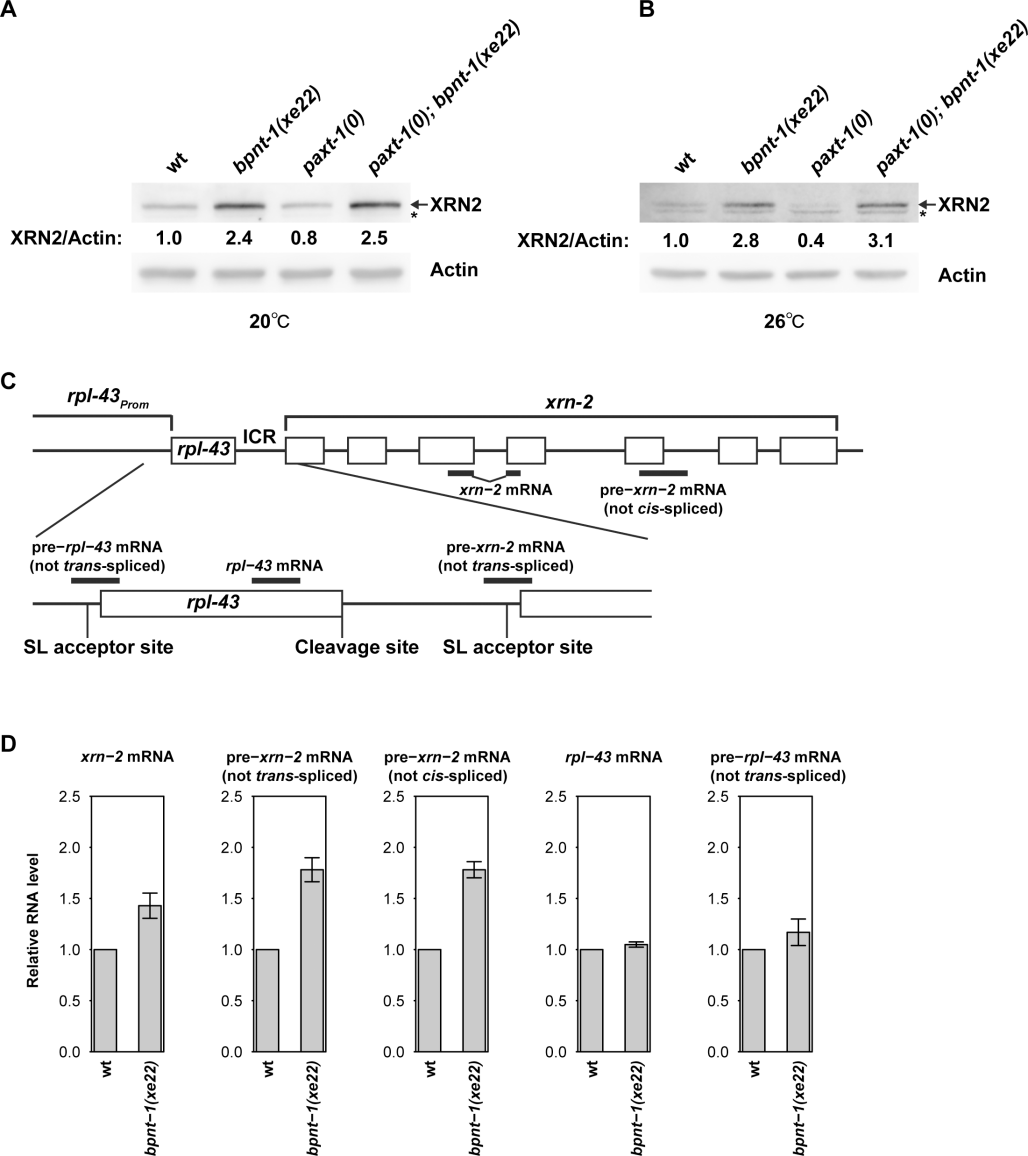
BPNT1 depletion increases *xrn-2* mRNA levels without stimulating its promoter activity. (A, B) wt, *bpnt-1(xe22)*, *paxt-1(0)*, and *paxt-1(0); bpnt-1(xe22)* animals were cultured from L1 to L4 for 40 hours at 20°C (A) or 30 hours at 26°C (B) before levels of XRN2 and Actin were detected by Western blot. XRN2 levels were normalized to actin levels and shown with values of wt defined as 1.0. Asterisks indicate unspecific bands. (C) Schematic representation of the *rpl-43~xrn-2* operon and the qPCR amplicons. Exons are shown as boxes, and qPCR amplicons as thick lines. SL acceptor sites and the 3‘ end cleavage site of *rpl-43* are indicated. (D) wt or *bpnt-1(xe22)* animals were cultured from L1 to L4 for 40 hours at 20°C. Levels of indicated pre-mRNA or mRNA were quantified by RT-qPCR and normalized to *act-1* mRNA levels with wild-type values defined as 1 (n = 3, means ± SEM). Values are shown in S1 Table.

A major substrate of BPNT1 proteins is PAP, which inhibits activity of XRN2 [16]. Therefore, in a simple model, loss of BPNT1 function would promote inhibition of XRN2, and thus enhance rather than suppress the larval arrest phenotype caused by XRN2 depletion in the absence of PAXT-1. To understand the discrepancy between the model and the data, we examined whether XRN2 levels were altered in the *paxt-1(0); bpnt-1(xe22)* animals. XRN2 protein signal intensity was increased more than two-fold in both *bpnt-1(xe22)* and *paxt-1(0); bpnt-1(xe22)* animals as compared to wild-type animals at both normal and elevated temperatures, while it was reduced in *paxt-1(0)* animals as previously reported [23] (Fig 2A and 2B and S3 Fig). Thus, increased XRN2 levels upon BPNT1 depletion may prevail over a putative decrease in specific enzymatic activity. As this effect occurs independently of the presence of PAXT-1 and independently of temperature, subsequent experiments examined the effect of *bpnt-1* mutation in *paxt-1(+)*, i.e., wild-type, animals at 20°C.

In order to establish how BPNT1 depletion boosted accumulation of XRN2 proteins, we quantified *xrn-2* mRNA levels. We found them to be increased by approximately 40% in *bpnt-1(xe22)* animals relative to wild-type animals (Fig 2C and 2D), indicating that accumulation of XRN2 proteins in *bpnt-1(xe22)* animals results, at least in part, from an increase in its mRNA levels.

The *xrn-2* gene is the downstream gene in a two-gene operon (WormBase ID: CEOP2697), where *rpl-43* is the upstream (promoter-proximal) gene. However, transcriptional upregulation of the operon does not appear to account for the increase of XRN2 in the *bpnt-1* mutant animals. This is because both mature mRNA and pre-mRNA levels of *rpl-43* were unaltered by *bpnt-1(xe22)*. (Fig 2C and 2D; note that because pre-*rpl-43* does not contain an intron, we utilized the fact that it contains an outron, which is removed by *trans*-splicing, to quantify it.) In striking contrast, however, we observed a consistent upregulation of pre-*xrn-2* mRNA levels with two sets of specific primers (Fig 2C and 2D): one detected pre-*xrn-2* mRNA that had not undergone *trans*-splicing, the other detected pre-*xrn-2* mRNA that had not undergone *cis*-splicing, i.e., still contained an intron. Levels of both pre-mRNA products were increased by approximately 70% in *bpnt-1(xe22)* animals as compared to wild-type animals (Fig 2C and 2D). Thus, upregulation of *xrn-2* mRNA upon BPNT1 depletion occurs, at least in part, at pre-mRNA level prior to *trans*-splicing, but neither through transcriptional activation nor stabilization of the *rpl-43*_*xrn-2* polycistronic transcript.

### BPNT1 depletion induces XRN2 de-repression

**Fig 3:**
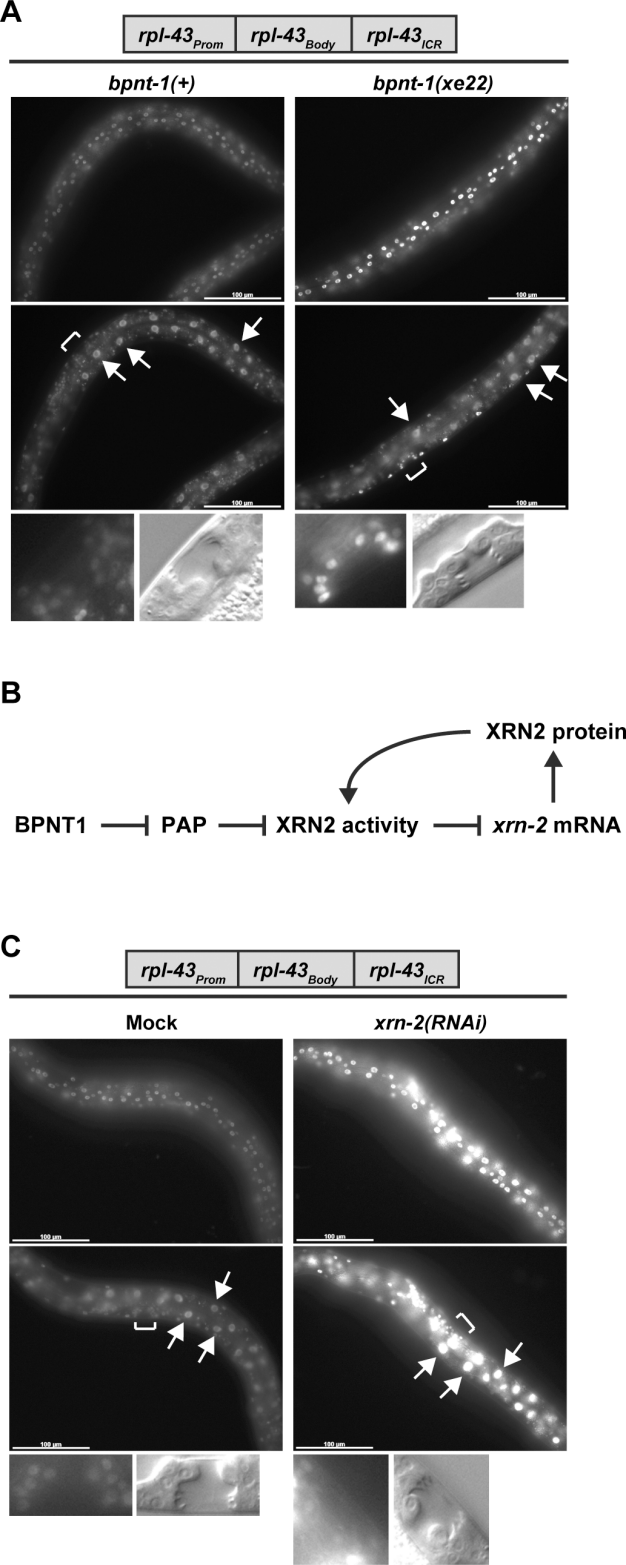
BPNT1 depletion induces XRN2 de-repression. (A) *rpl-43*_*Prom*_::*rpl-43*_*Body*_::*rpl-43*_*ICR*_ reporter animals in wild-type (*bpnt-1(+)*) or *bpnt-1(xe22)* genetic background were cultured from L1 to L4 for 40 hours at 20°C and observed. GFP signal was detected in hypodermal (top), intestinal (middle, arrows) and vulval (bottom) cells. Positions of vulvae are indicated by square brackets. Corresponding DIC images of mid-L4 stage vulvae are shown (bottom). Scale bar: 100 μm. (B) Diagram of an inferred BPNT1-XRN2 regulatory network. (C) *rpl-43*_*Prom*_::*rpl-43*_*Body*_::*rpl-43*_*ICR*_ reporter animals were exposed to mock or *xrn-2* RNAi from L1 to L4 at 20°C and observed. GFP signal was detected in hypodermal (top), intestinal (middle, arrows) and vulval (bottom) cells. Images are shown as described in (A).

To test whether the *xrn-2* gene body or its 3’ untranslated region (UTR) was dispensable for XRN2 regulation, we created a reporter construct that contained the promoter of *rpl-43*~*xrn-2* operon (*rpl-43*_*Prom*_) followed by the *rpl-43* gene body (*rpl-43*_*Body*_) and the *rpl-43*_*ICR*_. A sequence encoding green fluorescent protein (GFP) and the nuclear protein histone H2B with the 3’ UTR of the unrelated *unc-54* gene was fused to the construct, generating *rpl-43*_*Prom*_::*rpl-43*_*Body*_::*rpl-43*_*ICR*_::*GFP*::*H2B*::*unc-54 3’ UTR*. This and all subsequent reporter transgenes in this study have *GFP*::*H2B*::*unc-54 3’ UTR* (which we will thus omit when referring to transgenes in the following) and were inserted at an intergenic genomic locus on chromosome V by MosSCI. *rpl-43*_*Prom*_::*rpl-43*_*Body*_::*rpl-43*_*ICR*_ promoted ubiquitous GFP expression as we reported previously [22], where the construct was called *Pxrn-2*. However, the *bpnt-1(xe22)* allele increased the GFP signal in hypodermal cells and in vulval cells (Fig 3A and Table 1). Thus *rpl-43*_*Prom*_::*rpl-43*_*Body*_::*rpl-43*_*ICR*_ is sufficient to recapitulate *xrn-2* upregulation upon BPNT1 depletion.

**Table 1:** Effects of *xrn-2* knockdown or the *bpnt-1(xe22)* allele on reporter activities.

Reporter	Treatment	Tissue		
		Hypodermis	Intestine	Vulva
*rpl-43*_*Prom*_::*rpl-43*_*Body*_::*rpl-43*_*ICR*_	*xrn-2(RNAi)*	+	+	-
	*bpnt-1(xe22)*	+	-	+
	*xrn-2(xe34)*	+	+	+
*rpl-43*_*Body*_::*rpl-43*_*ICR*_	*xrn-2(RNAi)*	+	+	-
	*bpnt-1(xe22)*	-	-	-
*rpl-43*_*ICR*_	*xrn-2(RNAi)*	+	+	-
	*bpnt-1(xe22)*	-	-	-
	*xrn-2(xe34)*	+ (26°C)	-	-
*ran-4*_*Prom*_::*ran-4*_*Body*_::*ran-4*_*ICR*_	*xrn-2(RNAi)*	-	-	-
	*bpnt-1(xe22)*	-	-	-
*rpl-43*_*Prom*_::*rpl-43*_*Body*_::*ran-4*_*ICR*_	*xrn-2(RNAi)*	-	-	-
	*bpnt-1(xe22)*	-	-	-
*ran-4*_*Prom*_::*ran-4*_*Body*_::*rpl-43*_*ICR*_	*xrn-2(RNAi)*	+	+	-
	*bpnt-1(xe22)*	-	-	+
*ran-4*_*Prom*_::*ran-4*_*Body*_::*cri-3*_*ICR*_	*xrn-2(RNAi)*	+	+	+
	*bpnt-1(xe22)*	-	-	+

Effects of *xrn-2(RNAi)*, *bpnt-1(xe22)*, or *xrn-2(xe34)* on reporter activities in indicated tissues are summarized. +: upregulated; -: no obvious change.

The finding that loss of BPNT1 activity caused upregulation of *xrn-2*, while, presumably, decreasing XRN2’s enzymatic activity by enabling a build-up of inhibitory PAP, made us consider that *xrn-2* mRNA accumulation was a direct consequence of inhibition of XRN2 activity (Fig 3B). In support of this notion, we previously found that XRN2 inactivation led to accumulation of its mRNA [22]. Moreover, depletion of *xrn-2* by RNA interference (RNAi) upregulated the *rpl-43*_*Prom*_::*rpl-43*_*Body*_::*rpl-43*_*ICR*_ reporter as evidenced by an enhanced GFP signal in hypodermal cells (Fig 3C and Table 1). In contrast to BPNT1 depletion, XRN2 depletion also activated the reporter in intestinal cells (Fig 3C and Table 1), presumably reflecting differences in extents and kinetics of XRN2 inactivation through RNAi-mediated *xrn-2* mRNA depletion versus *bpnt-1* mutation in different tissues. Collectively, the data indicate that XRN2 autoregulates, and we propose that *bpnt-1* mutation may achieve XRN2 upregulation through this circuit.

### XRN2 depletion activates a cryptic promoter in the ICR between *rpl-43* and *xrn-2*

**Fig 4:**
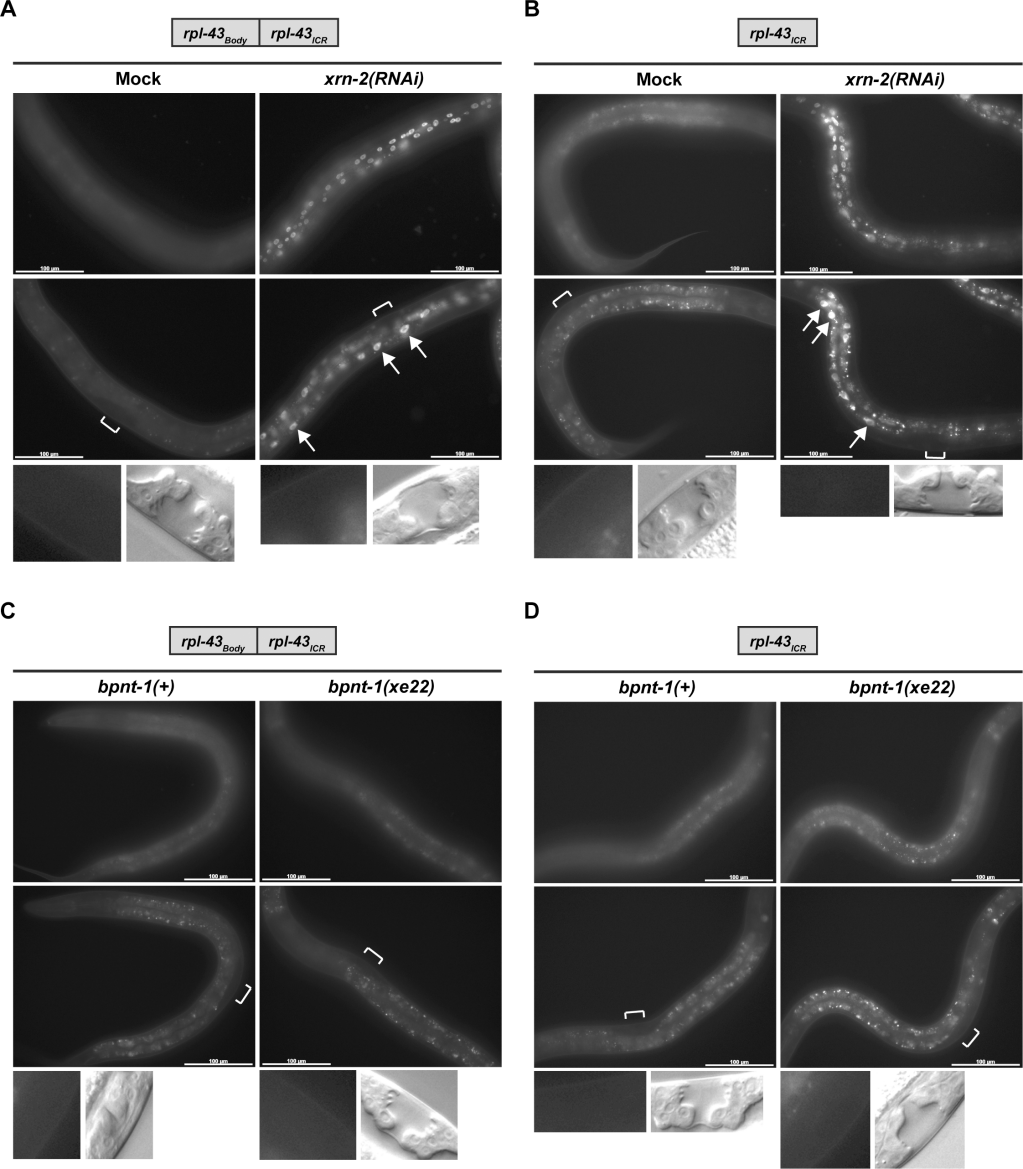
XRN2 depletion activates a cryptic promoter in the ICR between *rpl-43* and *xrn-2*. (A, B) Indicated reporter animals were exposed to mock or *xrn-2* RNAi from L1 to L4 at 20°C and observed. Upon XRN2 depletion, GFP signal was detected in hypodermal (top) and intestinal (middle, arrows) cells but not in vulval cells (bottom). Positions of vulvae are indicated by square brackets. Corresponding DIC images of mid-L4 stage vulvae are shown (bottom). Scale bar: 100 μm. (C, D) Indicated reporter animals in *bpnt-1(+)* or *bpnt-1(xe22)* genetic background were cultured from L1 to L4 at 20°C and observed. No signal was detected in hypodermal (top), intestinal (middle) or vulval (bottom) cells. Images are shown as described in (A).

Since pre-*xrn-2* mRNA levels increase in *bpnt-1(xe22)* in the apparent absence of increased *rpl-43*_*Prom*_ operon promoter activity, we wondered if *rpl-43*_*ICR*_ might exhibit promoter activity. To test this, we utilized a reporter construct, *rpl-43*_*Body*_::*rpl-43*_*ICR*_, which lacked *rpl-43*_*Prom*_. As expected, this reporter did not show detectable GFP signal in untreated animals. However, XRN2 depletion induced GFP expression in the hypodermis and the intestine (Fig 4A and Table 1). This XRN2-sensitive activity was independent of *rpl-43*_*Body*_, as the *rpl-43*_*ICR*_ reporter, which contains only *rpl-43*_*ICR*_, yielded comparable results (Fig 4B and Table 1). Thus, there is a cryptic promoter in *rpl-43*_*ICR*_, which is silent under normal conditions, but activated upon XRN2 depletion.

### A second mechanism for XRN2 autoregulation depends on the *rpl-43*_*ICR*_ but not cryptic promoter activity

Although the above results establish a cryptic promoter in *rpl-43*_*ICR*_ as a mechanism of XRN2 autoregulation, surprisingly, this does not appear to be the mechanism through which BPNT1 modulates XRN2 levels. This is because BPNT1 depletion did not induce detectable GFP expression from either of the reporters, *rpl-43*_*Body*_::*rpl-43*_*ICR*_ or *rpl-43*_*ICR*_ (Fig 4C and 4D). Given that BPNT1 depletion upregulates the *rpl-43*_*Prom*_::*rpl-43*_*Body*_::*rpl-43*_*ICR*_ reporter (Fig 3A), there must be an another regulatory mechanism that requires transcription from an operon promoter and does not induce cryptic promoter activity.

**Fig 5:**
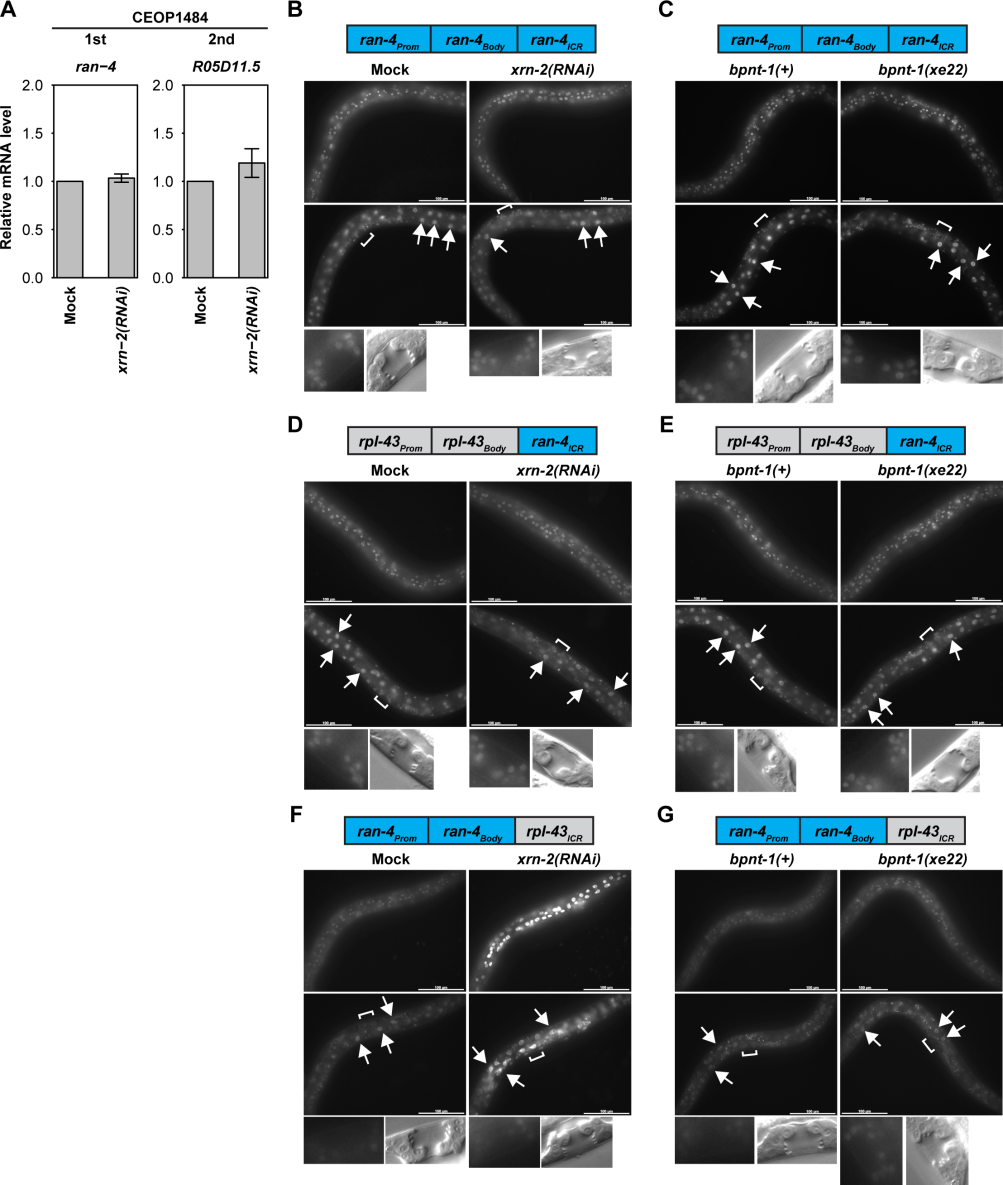
*rpl-43*_*ICR*_ is required while the operon promoter and the first gene are replaceable for XRN2 autoregulation. (A) wild-type animals were exposed to mock or *xrn-2* RNAi from L1 to L4 at 20°C. mRNA levels of *ran-4* and *R05D11*.*5* were quantified by RT-qPCR and normalized to *act-1* mRNA levels with mock values defined as 1 (n = 3, means ± SEM). Values are shown in S1 Table. (B, D, F) Indicated reporter animals were exposed to mock or *xrn-2* RNAi from L1 to L4 at 20°C and observed. GFP signal was detected in hypodermal (top), intestinal (middle, arrows) and vulval (bottom) cells. Positions of vulvae are indicated by square brackets. Corresponding DIC images of mid-L4 stage vulvae are shown (bottom). Scale bar: 100 μm. (C, E, G) Indicated reporter animals in *bpnt-1(+)* or *bpnt-1(xe22)* genetic background were cultured from L1 to L4 at 20°C and observed. GFP signal was detected in hypodermal (top), intestinal (middle, arrows) and vulval (bottom) cells. Images are shown as described in (B).

In order to determine which element of *rpl-43*_*Prom*_::*rpl-43*_*Body*_::*rpl-43*_*ICR*_ is required for the second regulatory mechanism, we performed element-swapping assays. To this end, we selected the *ran-4~F43G9*.*13* operon (WormBase ID: CEOP1484), which appears unaffected by XRN2 depletion. Specifically, mRNA levels for the first two genes of this eight-gene operon are comparable for *xrn-2(RNAi)* and mock RNAi animals (Fig 5A). A reporter that consisted of the operon promoter (*ran-4*_*Prom*_), the *ran-4* gene body (*ran-4*_*Body*_) and the ICR between the first and the second genes (*ran-4*_*ICR*_) induced GFP expression in many cell types including those of hypodermis, vulva and intestine, and, as expected, neither XRN2 depletion nor the *bpnt-1(xe22)* allele had obvious effects on the expression (Fig 5B and 5C and Table 1).

When we replaced *rpl-43*_*ICR*_ of the *rpl-43*_*Prom*_::*rpl-43*_*Body*_::*rpl-43*_*ICR*_ reporter by *ran-4*_*ICR*_, XRN2 autoregulation was abrogated: Neither depletion of XRN2 by RNAi nor mutation of *bpnt-1* caused an increase in GFP expression (Fig 5D and 5E and Table 1), suggesting that *rpl-43*_*ICR*_ is necessary for both autoregulatory mechanisms. To determine whether this element was also sufficient for autoregulation, we generated a *ran-4*_*Prom*_::*ran-4*_*Body*_::*rpl-43*_*ICR*_ reporter. This reporter showed markedly reduced GFP expression in a wild-type situation (Fig 5F) relative to the *ran-4*_*Prom*_::*ran-4*_*Body*_::*ran-4*_*ICR*_ reporter (Fig 5B), suggesting that *rpl-43*_*ICR*_ may reduce downstream transcript levels. XRN2 depletion enhanced GFP expression of the reporter in hypodermal and intestinal cells (Fig 5F and Table 1). Hence, *rpl-43*_*ICR*_ is both necessary and sufficient for XRN2 autoregulation.

To test specifically whether this ICR suffices to mediate also the cryptic promoter-independent mechanism utilized by BPNT1 for XRN2 regulation, we examined the effect of *bpnt-1* mutation. As shown in Fig 5G, BPNT1 depletion increased GFP expression in vulval cells. Consistent with the results from the *rpl-43*_*Prom*_::*rpl-43*_*Body*_::*rpl-43*_*ICR*_ reporter (Fig 3A), no obvious increase of GFP signal was observed in the intestine (Fig 5G and Table 1). Thus *rpl-43*_*ICR*_ is necessary and sufficient for both cryptic promoter-dependent and–independent XRN2 autoregulatory mechanisms. At the same time, the *bpnt-1* mutation causes strong hypodermal de-repression of *rpl-43*_*Prom*_::*rpl-43*_*Body*_::*rpl-43*_*ICR*_ but not *ran-4*_*Prom*_::*ran-4*_*Body*_::*rpl-43*_*ICR*_, possibly suggesting the participation of additional, context-dependent elements in different tissues, which remain to be identified.

### Two XRN2 repression mechanisms depend on different levels of XRN2 activity

**Fig 6:**
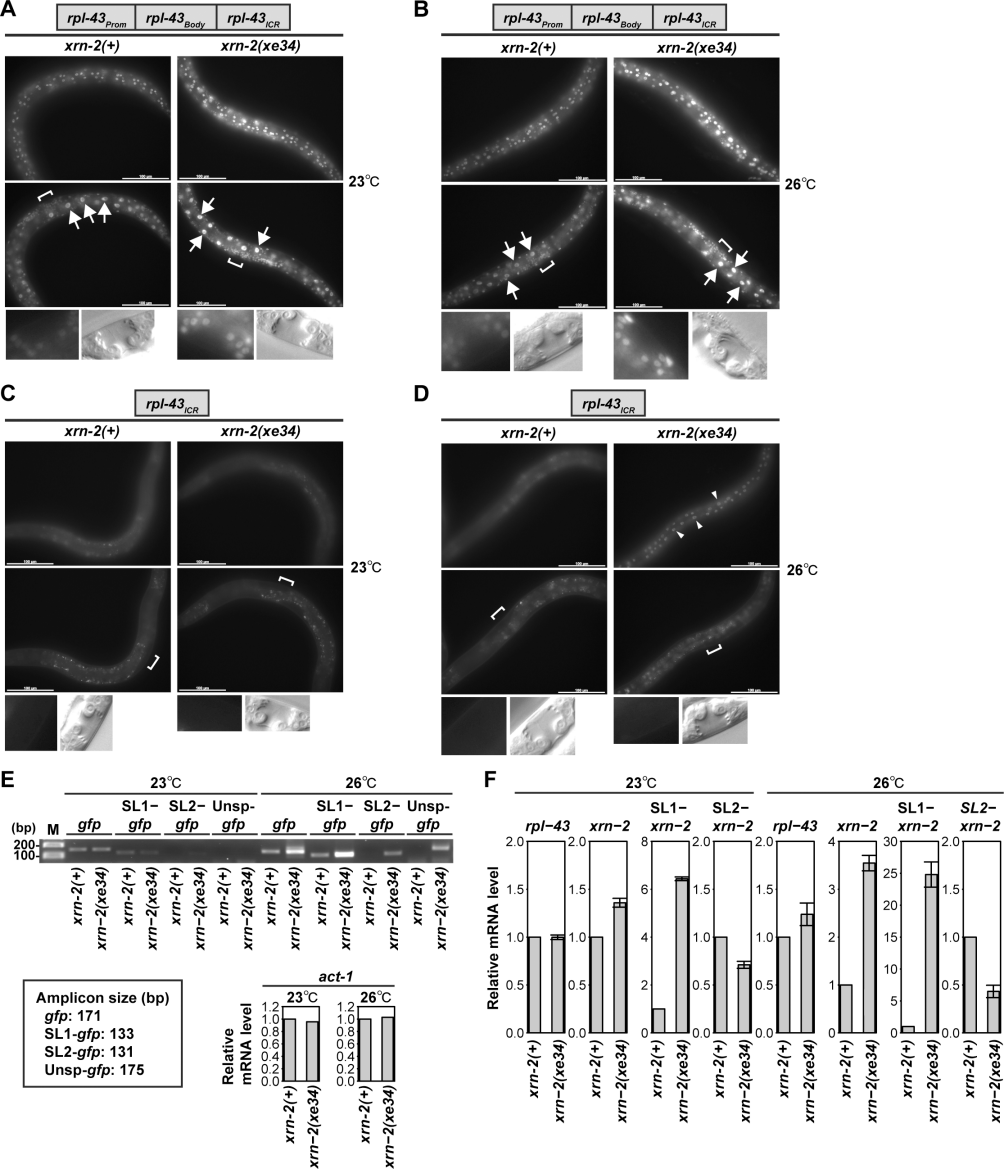
Two XRN2 repression mechanisms depend on different levels of XRN2 activity. (A-D) Indicated reporter animals in *xrn-2(+)* or *xrn-2(xe34)* genetic background were cultured from L1 to L4 at 23°C (A, C) or from L1 to L3 at 23°C followed by 26°C to L4 (B, D) and observed. Fluorescent images of hypodermal (top), intestinal (middle) and vulval (bottom) cells and corresponding DIC images of mid-L4 stage vulvae (bottom) are shown. Positions of vulvae are indicated by square brackets. Scale bar: 100 μm. (E, F) *rpl-43*_*ICR*_ reporter animals in *xrn-2(+)* or *xrn-2(xe34)* genetic background were cultured from L1 to L4 at 23°C and 26°C in the same way as (A) and (B), respectively. Images are shown as described in (A). Arrowheads indicate hypodermal signal. (E) Indicated *gfp* mRNAs were detected by RT-PCR with 40 cycles of amplification followed by agarose gel electrophoresis. Unsp: not *trans*-spliced, M: molecular weight marker. Amplicon sizes are shown below. Comparable levels of *act-1* mRNA in the template RNA were detected by RT-qPCR. (F) Levels of indicated endogenous mRNA species were quantified by RT-qPCR and normalized to *act-1* mRNA levels with values of *xrn-2(+)* animals defined as 1 (n = 3, means ± SEM). Values are shown in S1 Table.

The cryptic promoter in the *rpl-43*_*ICR*_ is de-repressed by *xrn-2* RNAi but not by BPNT1 depletion (Fig 4). Given that *xrn-2* RNAi causes developmental phenotypes such as slow growth [28] and a molting defect [29] while *bpnt-1(xe22)* animals show no obvious phenotype, we speculated that the cryptic promoter is de-repressed when XRN2 activity is severely reduced. To test this, we used the *xrn-2* temperature-sensitive allele, *xrn-2(xe34)* (S1 Fig). The *xrn-2(xe34)* allele increased GFP signal of the *rpl-43*_*Prom*_::*rpl-43*_*Body*_::*rpl-43*_*ICR*_ reporter in hypodermal, intestinal and vulval cells as compared to wild-type *xrn-2* (*xrn-2(+)*) both at 23°C (Fig 6A) and 26°C (Fig 6B). On the other hand, it activated the cryptic promoter in the *rpl-43*_*ICR*_ in hypodermal cells at 26°C (Fig 6D) but not at 23°C (Fig 6C). These results indicate that the *xrn-2(xe34)* allele promotes cryptic promoter-independent accumulation of *xrn-2* both at 23°C and 26°C, while it activates the cryptic promoter only at 26°C. To confirm that the cryptic promoter is inactive at 23°C, we examined *gfp* mRNA transcribed from the *rpl-43*_*ICR*_ by RT-qPCR. Since we failed to quantify its levels due to little or no expression in the presence of wild-type *xrn-2*, we examined its expression by RT-PCR (Fig 6E). At 23°C, *gfp* mRNA showed weak signal without a substantial difference between *xrn-2(+)* and *xrn-2(xe34)*. At 26°C, on the other hand, *gfp* mRNA showed elevated expression in the *xrn-2(xe34)* background. Thus, the two XRN2 repression mechanisms are alleviated at different thresholds of XRN2 activity, where activation of the cryptic promoter requires more severe reduction of XRN2 activity. Failed activation of the cryptic promoter in the intestine by *xrn-2(xe34)*, in contrast to *xrn-2* RNAi (Fig 4B), might be due to insufficient XRN2 inactivation by *xrn-2(xe34)* and/or very efficient intestinal XRN2 depletion through RNAi by feeding.

It has been reported that non-promoter-proximal genes in operons obtain a cap at the 5’ end of their mRNA mainly by *trans*-splicing to the spliced leader RNA SL2 [1]. However, this spliced leader selection is not exclusive. A small portion of their mRNA is *trans*-spliced to SL1, and the proportion increases particularly for those that have an internal promoter in their upstream ICRs [5]. In order to see which spliced leader of *xrn-2* mRNA accumulates upon the cryptic promoter-independent XRN2 de-repression, we examined *xrn-2(xe34)* animals cultured at 23°C. While *rpl-43* mRNA showed no change, *xrn*-2 mRNA levels increased approximately 40% (Fig 6F). Surprisingly, despite the SL2 preference of downstream operonic genes, the 40% increase was a result of a 6-fold increase of SL1-*xrn-2* mRNA and a 30% reduction of SL2-*xrn-2* mRNA (Fig 6F). The SL2-to-SL1 shift proceeded further at 26°C, namely, a 25-fold increase of SL1-*xrn-2* mRNA and a 60% reduction of SL2-*xrn-2* mRNA resulted in a 3.5-fold increase of *xrn-2* mRNA (Fig 6F). Although the cryptic promoter was de-repressed in this condition and hence partially responsible for the increase of SL1-*xrn-2* mRNA, further reduction of SL2- *xrn-2* mRNA was likely to be a result of the other mechanism (Fig 6E and 6F).

### XRN2 controls polycistronic gene expression of a subset of operons

**Fig 7:**
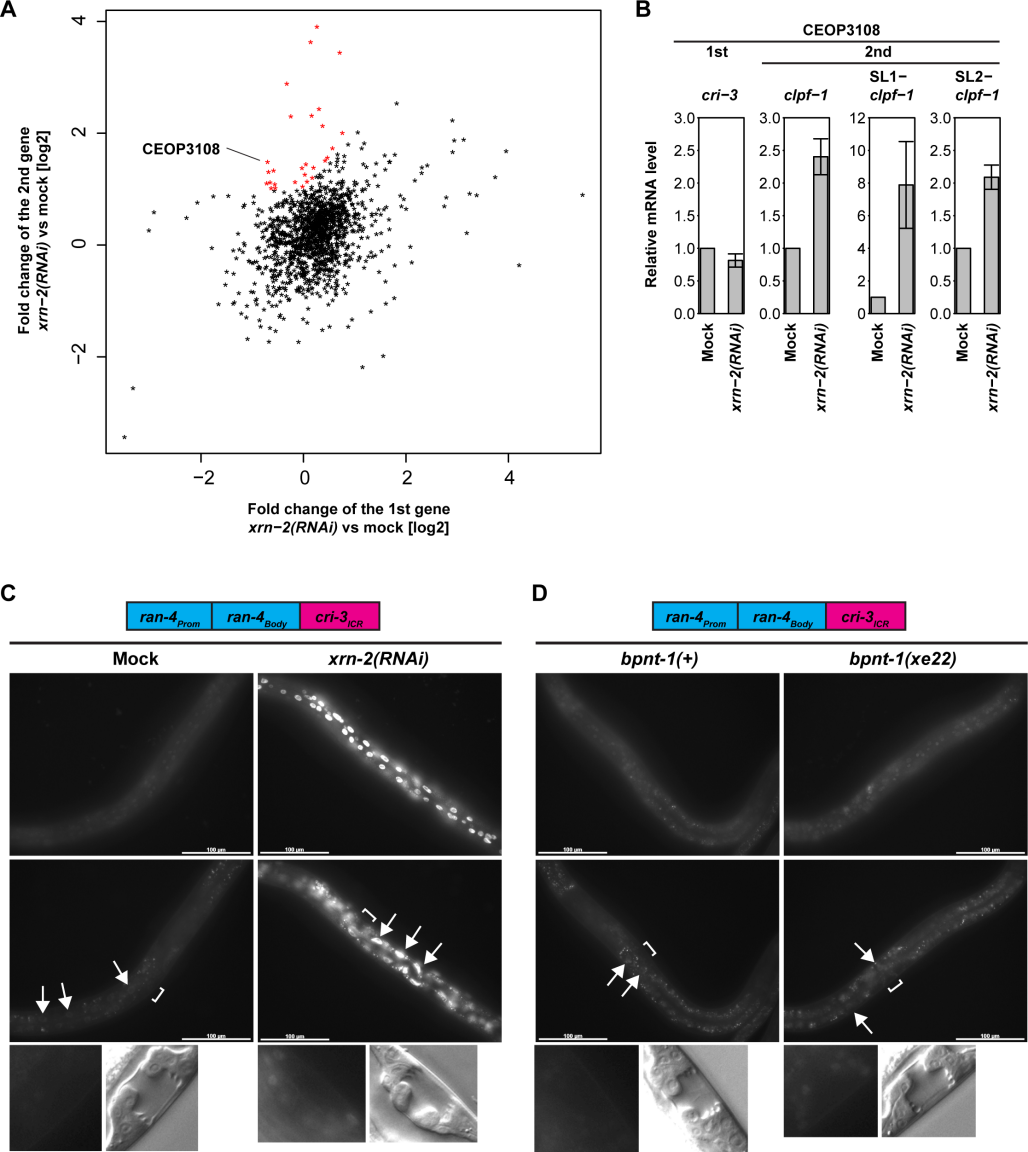
XRN2 controls polycistronic gene expression of a subset of operons. (A) wild-type animals were exposed to mock or *xrn-2* RNAi from L1 to L4 at 20°C. RNA was extracted, and poly(A)-RNA expression was examined by deep sequencing. Relative mRNA levels of the first and the second genes in operons were quantified. Fold changes of the second gene mRNA levels upon *xrn-2* RNAi are plotted against those of the first gene mRNA levels. Operons whose second gene mRNA showed a larger fold change than the first gene mRNA (cut-off as described in “Materials and Methods”) are shown in red. (B) wt animals were exposed to mock or *xrn-2* RNAi from L1 to L4 at 20°C. mRNA levels were quantified by RT-qPCR and normalized to *act-1* mRNA levels with control values defined as 1 (n = 3, means ± SEM). Values are shown in S1 Table. (C) *ran-4*_*Prom*_::*ran-4*_*Body*_::*cri-3*_*ICR*_ rerporter animals were exposed to mock or *xrn-2* RNAi from L1 to L4 at 20°C and observed. GFP signal was detected in hypodermal (top), intestinal (middle, arrows) but not in vulval (bottom) cells. DIC images of mid-L4 stage vulvae are shown (bottom). Scale bar: 100 μm. (D) *ran-4*_*Prom*_::*ran-4*_*Body*_::*cri-3*_*ICR*_ reporter animals in *bpnt-1(+)* or *bpnt-1(xe22)* genetic background were cultured from L1 to L4 at 20°C and observed. GFP signal was detected in hypodermal (top), intestinal (middle, arrows) and vulval (bottom) cells. Positions of vulvae are indicated by square brackets. Corresponding DIC images of mid-L4 stage vulvae are shown (bottom). Scale bar: 100 μm.

To see whether XRN2 regulates other operons in a similar way, we examined the effects of XRN2 depletion on operon gene expression globally by poly(A)-RNA sequencing (GEO ID: GSE79994; http://www.ncbi.nlm.nih.gov/geo/query/acc.cgi?token=kfwjugyizrkhdgt&acc=GSE79994). To identify XRN2-sensitive operons, we calculated fold changes for each gene upon *xrn-2 versus* mock RNAi, and plotted these for the second against the first gene for the 1388 annotated operons (Wormbase release: WS249) (Fig 7A, S5 Fig and S2 Table). Although this failed to provide a clear separation of XRN2-sensitive from XRN-2-insensitive operons, a subset of operons showed a greater extent of upregulation in the second gene than the first gene.

Among the 27 XRN2-sensitive operons, 9 had relatively short (<150 nt) ICRs like the *rpl-43~xrn-2* operon (S2 Table). Since operons with long ICRs might show XRN2-sensitivity solely through activation of an internal promoter, we focused on operons with short ICRs. Of those, we selected the *cri-3~clpf-1* operon (WormBase ID: CEOP3108) whose first and second genes showed relatively strong expression for further examination (S2 Table). Quantification of their mRNA levels by RT-qPCR recapitulated the changes upon *xrn-2* RNAi (Fig 7B) or *xrn-2(xe34)*-mediated XRN2 inactivation (S4 Fig). In contrast to the *rpl-43_xrn-2* operon, both SL1- and SL2-*xrn-2* mRNAs were upregulated.

In order to examine whether the ICR between *cri-3* and *clpf-1* (*cri-3*_*ICR*_) is responsible for the susceptibility to XRN2 depletion, we replaced *ran-4*_*ICR*_ of the *ran-4*_*Prom*_::*ran-4*_*Body*_::*ran-4*_*ICR*_ reporter by the ICR between *cri-3* and *clpf-1* (*cri-3*_*ICR*_). Like *rpl-43*_*ICR*_, *cri-3*_*ICR*_ reduced the GFP signal of the reporter in a wild-type situation (Fig 7C) relative to the *ran-4*_*Prom*_::*ran-4*_*Body*_::*ran-4*_*ICR*_ reporter (Fig 5B). Moreover, XRN2 depletion increased GFP expression in hypodermal, intestinal and vulval cells (Fig 7C and Table 1), and BPNT1 depletion did so in vulval cells (Fig 7D and Table 1). Hence, *cri-3*_*ICR*_ provides another instance of an ICR that confers XRN2-sensitivity to an operon downstream gene.

## Discussion

### An autoregulatory loop explains an unexpected genetic interaction between *xrn-2* and *bpnt-1*

Loss of BPNT homologues enhances or recapitulates phenotypes of XRN mutant yeast [16] and plants [18, 19, 20, 21]. This is consistent with its molecular function of hydrolyzing, and thus inactivating, PAP, an inhibitor of XRN2 catalytic activity [16, 17, 30]. By contrast, and unexpectedly, we show here that loss of *bpnt-1* function can suppress lethality due to decreased XRN2 activity in *paxt-1(0)* animals. Formally, we cannot exclude that *C*. *elegans* BPNT1 differs in function from its orthologues in other eukaryotes. However, we prefer an alternative interpretation, namely that these data reveal an unanticipated, non-linear behavior of the XRN2 pathway. Such behavior was not readily inferable from previous biochemical and other knowledge on the pathway’s components, thus highlighting the value of unbiased, phenotype-based genetic screens. The interpretation is consistent with, first, conservation of BPNT1’s function from yeast to mammals [17, 30], second, structural conservation between the rat and *C*. *elegans* protein, and, third, most importantly, our elucidation of XRN2 autoregulatory pathways that can account for this non-linear behavior. Thus, XRN2 functions to reduce *xrn-2* mRNA levels and, conversely, loss of XRN2 function increases *xrn-2* expression. This negative feedback autoregulation functions as a buffer to keep XRN2 accumulation within a certain range. As XRN2 has multiple and essential functions in RNA metabolism and development, robust maintenance of XRN2 levels may be important to protect the organism from sudden changes in environment. XRN2 autoregulation then works in concert with other mechanisms that ensure robustness of XRN2 activity, such as its stabilization by PAXT-1 [23, 24]. Finally, the specificities that we observe in extent, spatial pattern, and mechanism of XRN2 upregulation under different conditions (Table 1) suggests a broad utility of these pathways in buffering XRN2 activity against perturbations of different extents and dynamics.

### Mechanisms of polycistronic gene regulation by XRN2

**Fig 8:**
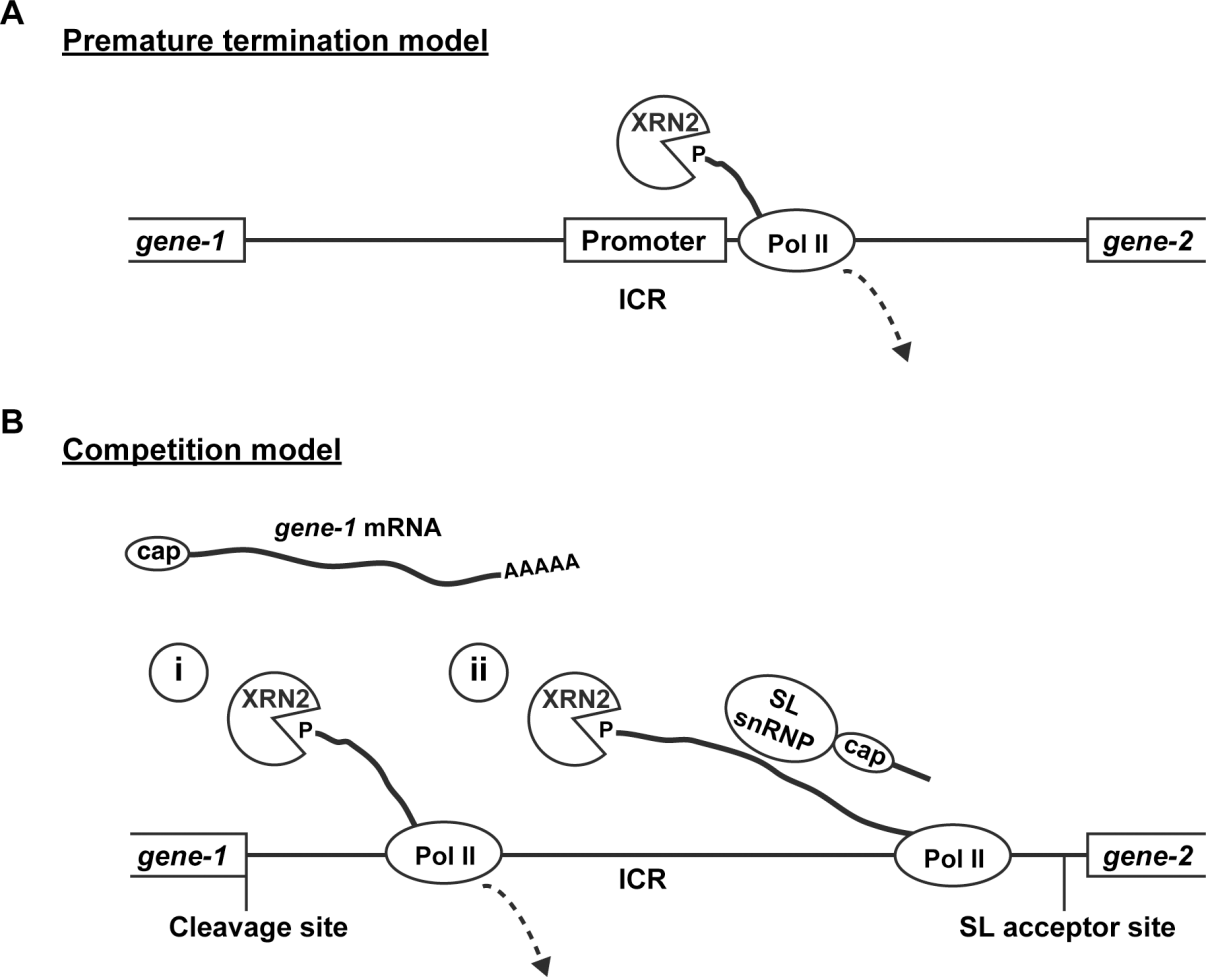
Mechanistic models for polycistronic gene regulation by XRN2. XRN2-mediated polycistronic gene regulation occurs through an (A) operon promoter-independent and (B) an operon-promoter-dependent mechanism. (A) Premature termination model. XRN2 degrades uncapped or decapped nascent RNA transcripts from a promoter in the ICR and terminates transcription at promoter proximal site. (B) Competition model. Following 3’ end cleavage of the upstream gene, XRN2 degrades the downstream fragment. Competition with (i) ongoing transcription by RNA pol II, whose activity XRN2 can terminate, or (ii) capping by *trans*-splicing determines production of *xrn-2* mRNA. SL indicates SL1 or SL2.

Autoregulation of XRN2 is facilitated, at least in part, by *xrn-2* being in an operon, as shown by the fact that we identify two mechanisms, both of which rely on the ICR between *xrn-2* and its upstream gene but on different thresholds of XRN2 activity for induction. The two mechanisms also differ with respect to their reliance on the upstream operon promoter. One mechanism can function in the absence of this promoter, suggesting that the ICR that separates *xrn-2* from *rpl-43*, although unusually short [5], contains a cryptic promoter the activity of which XRN2, directly or indirectly, counteracts. Based on published data from human cells, we may speculate on the underlying mechanism: Brannan *et al*. [31] reported human XRN2 to localize near the transcription start sites of some genes together with decapping proteins and the termination factor TTS2, and to terminate transcription by RNA polymerase II (Pol II) near promoter proximal sites. Based on these and additional observations, they proposed that XRN2 degrades a nascent transcript following decapping and dislodges Pol II from the DNA template. A similar mechanism might then repress transcription from the internal promoter of the *rpl-43*~*xrn-2* operon (Fig 8A).

The second mechanism of XRN2 autoregulation requires both the ICR and the operon promoter. In the reporter assay, the operon promoter could be replaced by another promoter, suggesting that promoter specificity is not crucial for the autoregulation. Moreover, BPNT1 depletion, which could alleviate only the operon promoter-dependent repression mechanism, increased pre-mRNA levels of *xrn-2* without affecting those of *rpl-43*. We can envision two, not mutually exclusive, scenarios that would explain these observations. Both of these involve the catalytic activity of XRN2 to degrade 5’ monophosphorylated RNA and its competition with other processes (Fig 8B): Once pol II transcribes past the polyadenylation site (PAS) of the first gene, *rpl-43*, pre-*rpl-43* mRNA is cleaved and polyadenylated at the 3’ end and separated from the polycistronic transcript. This cleavage leaves a monophosphate at the 5’ end of the downstream transcript, which could be an XRN2 substrate until it becomes protected through acquisition of a 5’ cap structure present on the spliced leader sequence. In the first scenario, XRN2-mediated degradation would compete with *trans*-splicing-mediated stabilization of the pre-mRNA. In the second scenario, the competition would occur between XRN2 and RNA pol II. In this model, the ICR would permit or even promote some degree of transcription termination downstream of *rpl-43*. In yeast and mammalian cells, XRN2 promotes termination by degrading the transcript downstream of the PAS until it reaches RNA pol II, which causes dissociation of the polymerase from the DNA template and thus cessation of transcription [32].

Either mechanism would explain both the reliance on an upstream operon promoter and the ICR. At this point, our data cannot distinguish between these two mechanisms, and, and it seems indeed possible that both might operate in parallel. Given the *trans*-splicing of downstream operon genes to SL2 [5], we would have expected, but did not observe, a preferential increase in the levels of SL2 rather than SL1 *trans*-spliced transcripts if competition with *trans*-splicing were the major mechanism. However, since the specificity of SL2 over SL1 seems not absolute [5], it seems premature to discount this model. At the same time, the termination competition model clearly appeals from the view of parsimony, as it suggests that both the operon promoter-dependent and -independent processes converge on the same molecular mechanism, namely competition between transcription by RNA pol II and its (premature) termination by XRN2.

### Diversifying operonic gene expression through XRN2

In addition to *xrn-2*, numerous genes in *C*. *elegans* occur in operons, although the functional relevance of this gene architecture is less clear: Unlike in prokaryotes, genes in a *C*. *elegans* operon are typically functionally unrelated, and their mRNA levels are not necessarily comparable [9]. XRN2 may contribute to uncoupling of gene expression patterns of genes sharing an operon, as we show for the *cri-3*~*clpf-1* operon. It will thus be important to achieve a clearer separation of XRN2-sensitive and -insensitive operons to quantify the magnitude of the effect in future work. Knowledge and characterization of additional instances may then also reveal how generalizable the underlying mechanisms are. At this point, regulation of both the *rpl-43*~*xrn-2* and the *cri-3*~*clpf-1* operons through ICRs indeed implies shared mechanisms.

## Materials and Methods

### Strains

The Bristol N2 strain was used as wild-type. The VC40114 strain isolated in the Million Mutation Project [26] was obtained from the Caenorhabditis Genetics Center (University of Minnesota, MN, USA). Strains used are shown in S3 Table.

### Worm culture

*C*. *elegans* worms were cultured on Nematode Growth Medium (NGM) agar seeded with *Escherichia coli* OP50 according to the standard methods described previously [33].

### Cloning and site-directed mutagenesis

Cloning and site-directed mutagenesis were performed by PfuUltra II Fusion HS DNA Polymerase (Agilent Technologies, Santa Clara, CA, USA) according to the supplier’s protocol using specific primers (S4 Table). PCR-amplified or synthesized (Integrated DNA Technologies, Coralville, IA, USA) DNA fragments were inserted to vectors by Gateway Technology (Life Technologies, Carlsbad, CA, USA) or Gibson Assembly [34].

### Single-copy transgene insertion

DNA fragments were inserted into the pCFJ150 vector by Multisite Gateway Technology (Life Technologies) according to the supplier’s protocol. *Mos1*-mediated Single-Copy transgene Insertion (MosSCI) was performed according to the previous report using the EG8082 strain [27].

### EMS mutagenesis and whole genome sequencing

About 6,000 L4-stage *paxt-1(xe5)* worms were harvested, washed and incubated with 50 mM EMS in 6 ml of M9 buffer for 4 hours at room temperature. The worms were washed three times with M9 buffer and cultured at 25°C. The L3- or L4-stage larvae of the F1 generation were cultured at 26°C, and their progeny were screened for normal development. One mutant line that was maintainable at 26°C was isolated and backcrossed 6 times with the parental *paxt-1(xe5)* strain to remove unrelated mutations. Genomic DNA was extracted and purified using Gentra Puregene Tissue Kit (Qiagen, Venlo, Netherlands). DNA libraries were created from 50 ng of genomic DNA using Nextera DNA Library Prep Kit (Illumina, San Diego, CA, USA). The sequencing data were generated using a HiSeq 2500 (Illumina).

### Processing of sequence data and detection of sequence variants

Sequence data were processed following a similar workflow as described previously [35]. Sequence reads were aligned to the May 2008 *C*. *elegans* assembly (obtained from http://hgdownload.soe.ucsc.edu/goldenPath/ce6/chromosomes/) using ‘‘bwa” [36] (version 0.7.4) with default parameters, but only retaining concordant single-hit alignments (“bwa sampe -a 1000 -o 1000 -n 1 -N 0” and selecting alignments with ‘‘X0:i:1”). The resulting alignments were converted to BAM format, sorted and indexed using ‘‘samtools” [37] (version 0.1.19). In order to quantify contamination by Escherichia coli, reads were similarly aligned to a collection of *Escherichia coli* (*E*. *coli*) genomes (NCBI accession numbers NC_008253, NC_008563, NC_010468, NC_004431, NC_009801, NC_009800, NC_002655, NC_002695, NC_010498, NC_007946, NC_010473, NC_000913 and AC_000091), which typically resulted in less than 1% aligned reads. Potential PCR duplicates were identified and removed using Picard (version 1.115, http://broadinstitute.github.io/picard/), reducing the number of reads to 93% to 95%. Sequence variants were identified using GATK [38] (version 3.1.1) following recommended “best practice variant detection”: Initial alignments were first corrected by indel realignment and base quality score recalibration, followed by SNP and INDEL discovery and genotyping using “UnifiedGenotyper” for each individual strain using standard hard filtering parameters, resulting in a total of ~10,000 sequence variations in each strain compared to the reference genome. Finally, high quality (score > = 200) variants not identified in the parent strain (n = 172) were checked for sequence support in the parent strain, resulting in a final set of 56 suppressor-strain specific variants. Of those 6 were clustered in a ~160 kb region on chromosome II (4,309,302–4,468,036), which contained a nonsense mutation in the *bpnt-1* gene.

### Microscopy

Stereoscopic images were obtained with an M205A stereo microscope (Leica, Solms, Germany). DIC and fluorescent images were obtained using an Axio Observer Z1 microscope and AxioVision SE64 (release 4.8) software (Carl Zeiss, Oberkochen, Germany). For GFP reporter assays, presence or absence of differences in signal intensity between conditions were evaluated by visual inspection of at least twenty worms. Where this revealed consistent patterns of difference, fluorescence and DIC Images of at least five randomly selected worms per condition were acquired for hypodermis, intestine, and vulva, and examined. In the experiments for Figs 4A, 4B and 6D, this confirmed that all observed worms were GFP-negative in control and GFP-positive in genetically modified conditions, respectively, in the tissues indicated “+” in Table 1. In the experiments for Figs 4C, 4D and 6C, all observed worms were GFP-negative in control and genetically modified conditions. In the experiments for Figs 3A, 3C, 5F, 6A, 6B, 7C and 7D, GFP was observed in both control and genetically modified conditions, but worms in genetically modified conditions consistently showed stronger GFP signal than worms in control conditions in the tissues indicated “+” in Table 1. In these instances, we specifically selected images of worms that showed the strongest GFP-signal in control and the weakest GFP-signal in genetically modified conditions, respectively, for comparison (i.e., we compared images where the difference between the conditions would be minimal). These images, shown in S6 Fig, confirmed robust differences. Multiple images of vulvae are shown in S6 Fig for the experiment for Fig 7D, where GFP signal was present but weak in the genetically modified condition. Finally, in the experiment for Fig 5G, the strongest GFP signal in the control condition was comparable to the weakest GFP signal in the genetically modified condition. Hence, we tested further whether a robust difference was observed by arranging images for each condition from strongest to weakest, which confirmed overall strong GFP signal for only the genetically modified condition (S6 Fig). In other experiments, no obvious differences in GFP signal intensity or tissue specificity were observed between control and genetically modified conditions.

### Western blotting

About 6,000 worms were harvested, washed three times with M9, resuspended in 100 μl of SDS lysis buffer (63 mM Tris-HCl (pH 6.8), 5 mM DTT, 2% SDS, 5% sucrose) and heated for 5 min at 95°C, followed by sonication. After centrifugation at 10,000 x g for 10 min at 4°C the supernatant was collected. 100 μg of the extract was subjected to SDS-PAGE and Western blot. A rat anti-XRN2 antibody [22], a mouse anti-Actin antibody (clone C4, Millipore, Billerica, MA, USA) and a mouse anti-FLAG antibody (clone M2, Sigma-Aldrich, St. Louis, MI, USA) were used with 1,000-, 3,000- and 1,000-fold dilutions, respectively, followed by horseradish peroxidase-conjugated secondary antibody (GE Healthcare, Little Chalfont, UK) reaction. The membranes were treated with ECL Western Blotting Detection Reagents, and protein bands were detected by an ImageQuant LAS 4000 chemiluminescence imager (all GE Healthcare). Band intensities were quantified using the ImageJ software (NIH, Bethesda, MD, USA).

### RNAi

The RNAi clone against *xrn-2* was obtained from the Ahringer library [39]. RNAi was performed by the feeding method [40]: bacteria carrying the insertless L4440 RNAi vector were used as a negative control. Since *xrn-2* RNAi causes slow growth, worms were treated with control or *xrn-2* RNAi from L1 to L4 stage for 40~42 or 48~52 hours, respectively, at 20°C. Vulval morphology was observed to confirm mid-L4 stage.

### RNA preparation, RT-qPCR and poly(A)-RNA sequencing

Worms were harvested, washed three times with M9 medium, resuspended in 1 ml of TRI Reagent (Molecular Research Center, Cincinnati, OH, USA) and frozen in liquid nitrogen. Worms were broken open by five repeats of freeze and thaw using liquid nitrogen and a 42°C heating block, before RNA was extracted and purified according to the supplier’s protocol with the modification that RNA was incubated with 50% 2-propanol at -80°C overnight for efficient precipitation. The purified RNA was treated with DNA-free Kit (Thermo Fischer Scientific, Waltham, MA, USA) to remove DNA. For mRNA quantification by RT-qPCR, cDNA was generated from total RNA by ImProm-II Reverse Transcription System (Promega, Fitchburg, WI, USA) using oligo(dT)_15_ primers (for mature mRNAs) or random primers (for pre-mRNAs) according to the supplier’s protocol. RT-qPCR was performed with specific primers (S4 Table), the SYBR Green PCR Master Mix (Applied Biosystems), and the StepOnePlus Real-time PCR System. After 40 cycles of PCR amplification, some samples were subjected to agarose gel electrophoresis (Fig 6F). For poly(A)-RNA sequencing, libraries were prepared using the TruSeq Standard mRNA Library Prep Kit (Illumina, San Diego, CA, USA) and sequenced.

### Operon gene expression analysis

In order to make use of the most recent operon annotations from WormBase, RNA-sequencing reads were aligned to the October 2010 (ce10) *C*. *elegans* assembly from UCSC [40]. Alignments were performed using the qAlign function from the QuasR R package [41], with the reference genome package (“Bsgenome.Celegans.UCSC.ce10”) downloaded from Bioconductor (https://www.bioconductor.org/) and setting the parameter “splicedAlignment = TRUE”, which calls the SpliceMap aligner with default parameters [42]. The resulting alignments were converted to BAM format, sorted and indexed using Samtools [37] (version 1.2). Expression was quantified on a gene level using annotations downloaded from WormBase (version WS220, which corresponds to the ce10 assembly) (ftp://ftp.wormbase.org/pub/wormbase/releases/WS220/species/c_elegans/) by counting reads overlapping all annotated exons for each gene. Samples were normalized by the mean number of counts mapping to all exons, and gene-level counts were log_2_-transformed after adding a pseudocount of 8. Operon annotations were downloaded from WormBase (version WS249) (ftp://ftp.wormbase.org/pub/wormbase/releases/WS249/species/c_elegans/PRJNA13758/), comprising 1,388 operons in total. For each operon, the log_2_ fold-changes in expression for *xrn-2* RNAi vs mock were calculated for both the first and second gene in the operon (S2 Table). These log_2_ fold-changes were then compared to identify operons in which the second gene was preferentially up-regulated compared to the first in *xrn-2* RNAi conditions, using the criteria that the second gene had to be at least 2-fold upregulated in RNAi vs mock and that the difference in fold-change between the second and the first gene had to be at least 2. All computations were performed using R (version 3.2.2) in the RStudio environment (version 0.99.484).

Extensive rhythmic gene expression during *C*. *elegans* development may impact the results of differential expression analysis, as false positives or false negatives may be introduced if experimental and control samples are not well-matched in developmental time [43]. To determine the timing of our samples, after sequencing two replicates each of *xrn-2* RNAi and mock and quantifying the gene expression levels in each, we calculated the Pearson correlation coefficient between each sample and a previously-sequenced mRNA timecourse sampling L4 larval development at 25°C at hourly intervals for 16 hours. We then evaluated the timepoint to which each of our samples showed the highest correlation. We found one pair of *xrn-2* RNAi and mock samples that were well-matched (replicate 1), with each showing the highest correlation to the 32h timepoint (*xrn-2* RNAi: r = 0.96, mock: r = 0.98); the second pair (replicate 2) were less well-matched and showed the highest correlations to the 35h (*xrn-2* RNAi: r = 0.94) and 36h (mock: r = 0.96) timepoints. We therefore focused our analysis, as described above, on the replicate 1 pair. We validated our initial results using the less well-matched pair by plotting the difference between the log fold-change of the second gene in operons and the log fold-change of the first gene in operons for each replicate against one another (S5 Fig). The correlation between the two replicates was 0.52; however, the majority of operons passing the cutoff for replicate 1, including the *cri-3~clpf-1* operon (CEOP3108), fell into the upper right quadrant of the plot, indicating that most of the expression changes were captured in both replicates.

## Supporting Information

S1 Fig *xrn-2* temperature-sensitive mutant. wt, *xrn-2(xe34)* or *xrn-2(xe34); xrn-2*::*gfp* animals were cultured from L1 at 20°C, 23°C or 26°C for 72 hours and observed by stereomicroscopy at the same magnification. *xrn-2(xe34)* animals developed normally to adults at 20°C while were arrested and died as larvae at 26°C. The temperature-sensitive phenotype of *xrn-2(xe34)* animals were rescued by expression of GFP-fused wild-type *xrn-2* (*xrn-2*::*gfp*).
(PDF)

S2 Fig Alignment of BPNT protein sequences. (A) Protein sequences of BPNT homologues in *S*. *cerevisiae* (Sc Hal2p), *R*. *norvegicus* (Rn Bpnt1) and *C*. *elegans* (Ce BPNT-1) are aligned by Clustal Omega (http://www.ebi.ac.uk/Tools/msa/clustalo/) and displayed by Jalview (http://www.jalview.org/).The most frequent amino acids are in blue, similar amino acids in light blue, based on BLOSUM62. Amino-acids numbers relative to the first methionine are shown. The *bpnt-1(xe22)* allele has a mutation that changes the 294th tryptophan to stop. (B) Structure of *R*. *norvegicus* Bpnt1. The corresponding region missing in the *C*. *elegans* BPNT-1(xe22/W294*) is shown in orange.
(PDF)

S3 Fig Western blot raw data. (PDF)

S4 Fig Effects of *xrn-2(xe34)* on the *cri-3_clpf-1* operon. *rpl-43*_*ICR*_ reporter animals in *xrn-2(+)* or *xrn-2(xe34)* genetic background were cultured from L1 to L3 at 23°C followed by 26°C to L4. Levels of indicated mRNA from the *cri-3_clpf-1* were quantified by RT-qPCR and normalized to *act-1* mRNA levels with values of *xrn-2(+)* animals defined as 1 (n = 3, means ± SEM). Values are shown in S1 Table.
(PDF)

S5 Fig Operon gene expression analysis. See Fig 7A and Materials and Methods. The difference in log2 fold-change between the second gene and the first gene in operons is plotted for each replicate against the other. Operons above the cut-off in replicate 1 are shown in red. The Pearson correlation coefficient between the fold-change differences for the two replicates is shown in the upper left.
(PDF)

S6 Fig Supporting data for GFP reporter assays. See Materials and Methods.
(PDF)

S1 Table RNA quantification data. (XLSX)

S2 Table Operon gene expression analysis data. See Materials and Methods. ICR lengths were determined based on annotated transcript start and end positions, which may not always be accurate, as reflected in negative numbers.
(XLSX)

S3 Table Strain information. (XLSX)

S4 Table Primer information. (XLSX)
