# Supplementary figures and images for: XRN2 Autoregulation and Control of Polycistronic Gene Expresssion in Caenorhabditis elegans
Source: PLoS Genet. 2016 Sep 15;12(9):e1006313. doi: 10.1371/journal.pgen.1006313 (PMC5025045; doi:10.1371/journal.pgen.1006313)

# S1 Figure

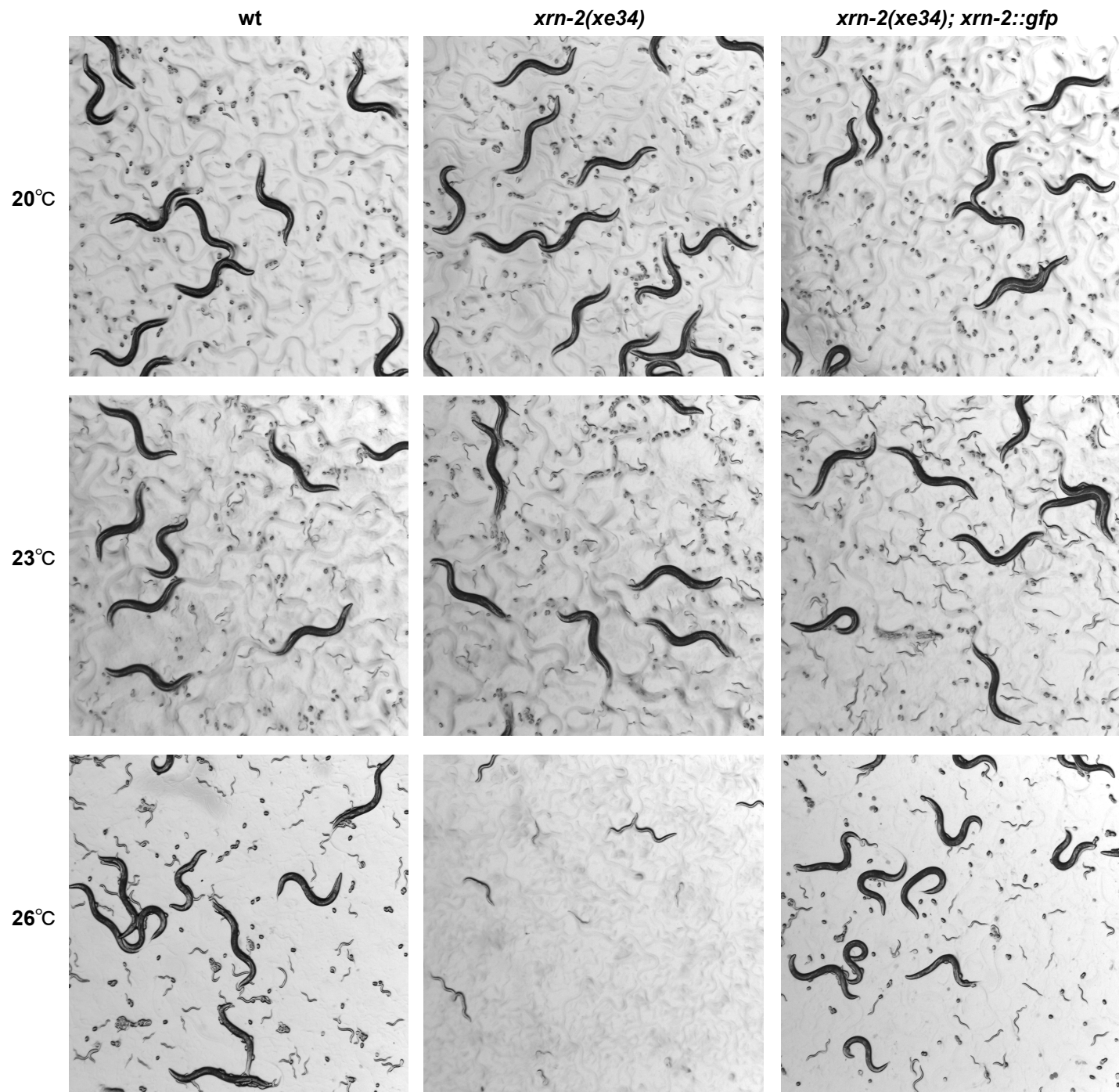

Supplement: S1 Fig — wt, xrn-2(xe34) or xrn-2(xe34); xrn-2::gfp animals were cultured from L1 at 20°C, 23°C or 26°C for 72 hours and observed by stereomicroscopy at the same magnification. xrn-2(xe34) animals developed normally to adults at 20°C while were arrested and died as larvae at 26°C. The temperature-sensitive phenotype of xrn-2(xe34) animals were rescued by expression of GFP-fused wild-type xrn-2 (xrn-2::gfp). (PDF) [file pgen.1006313.s001.pdf]

S3 Figure

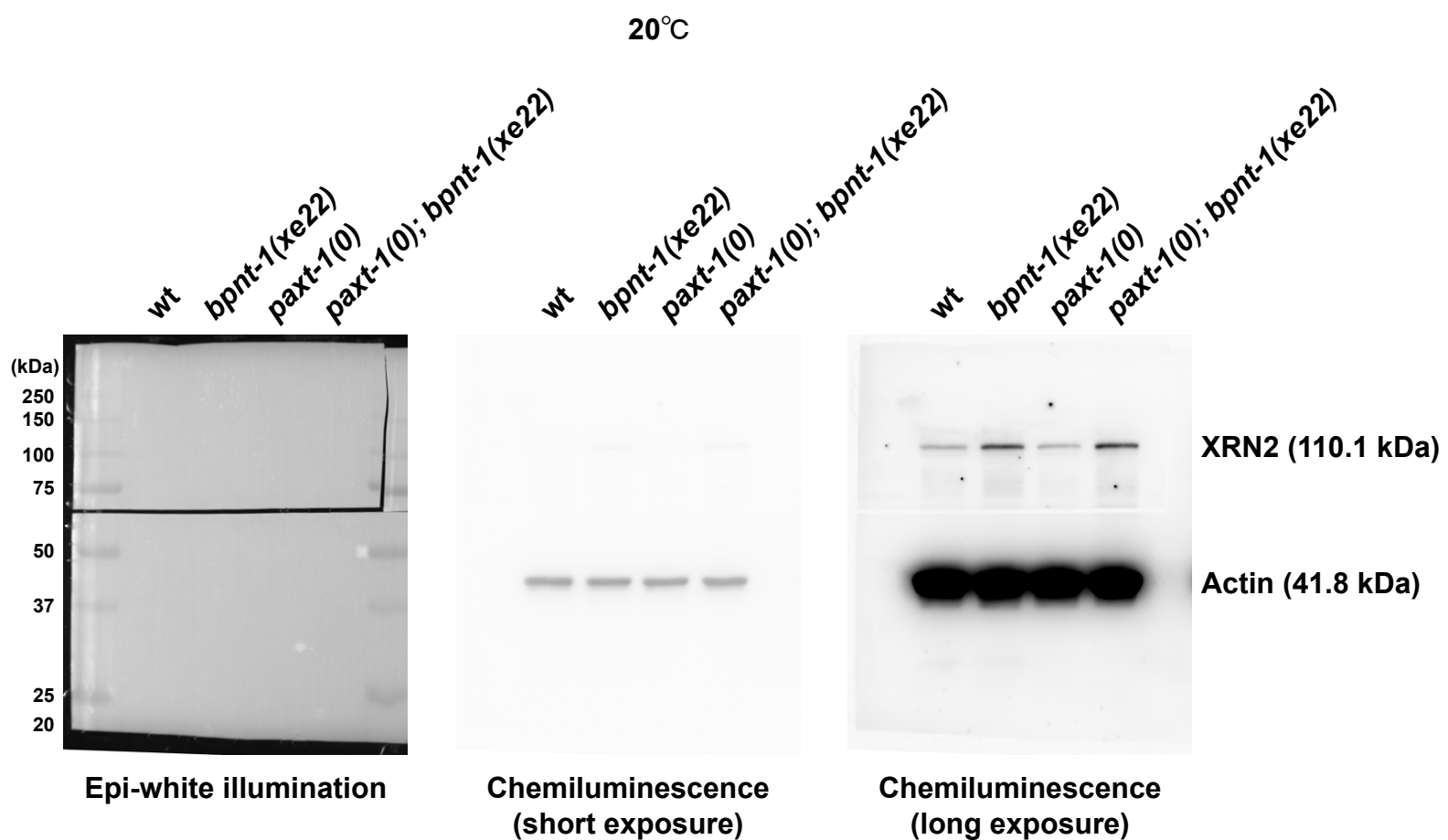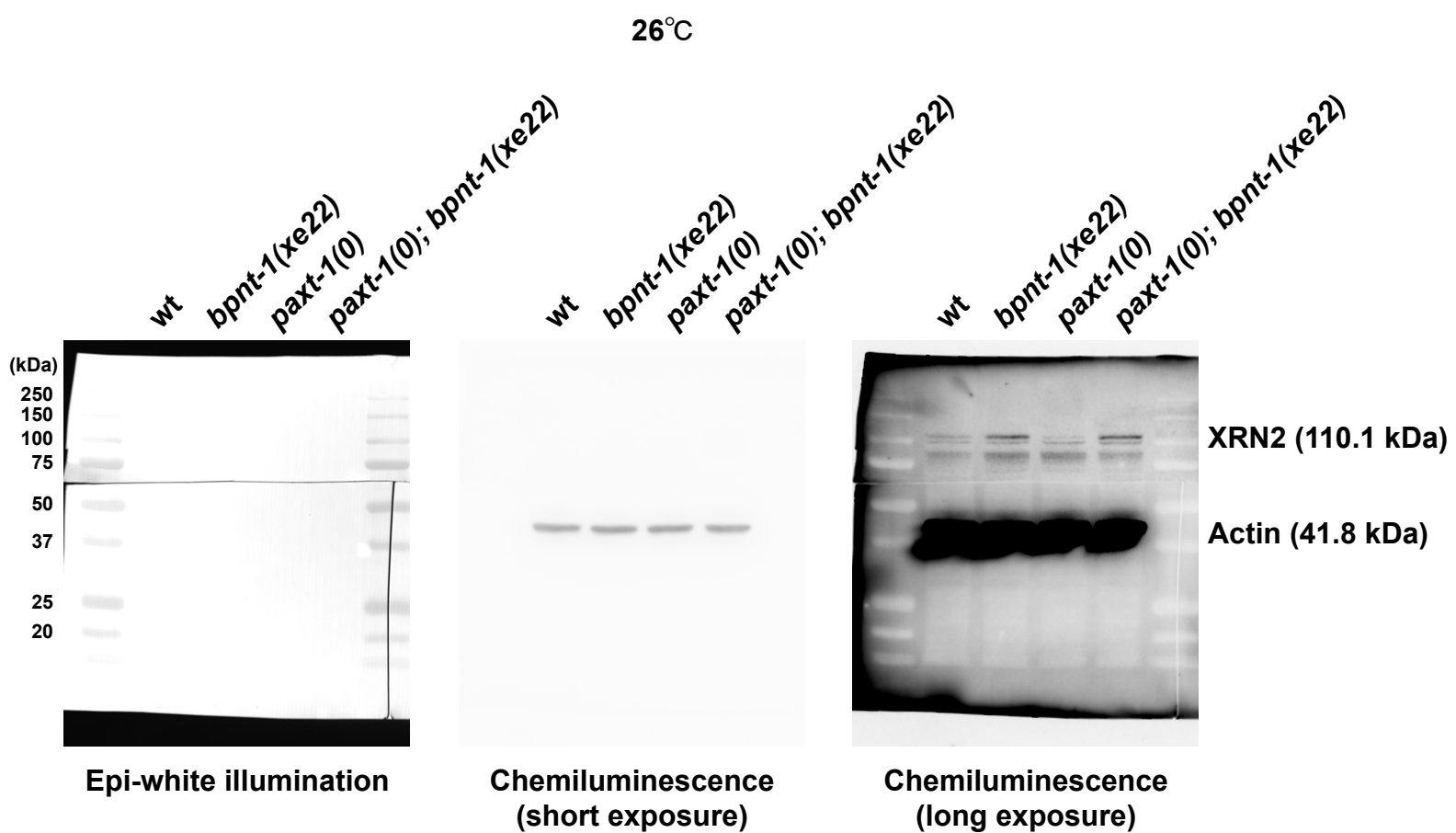

Supplement: S3 Fig — (PDF) [file pgen.1006313.s003.pdf]

S4 Figure

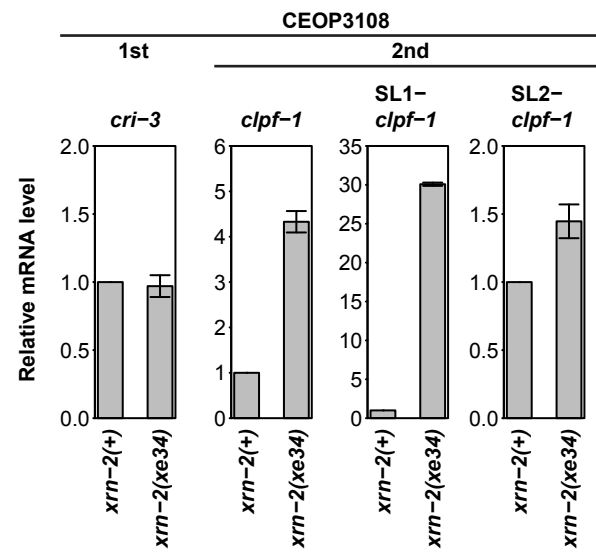

Supplement: S4 Fig — rpl-43ICR reporter animals in xrn-2(+) or xrn-2(xe34) genetic background were cultured from L1 to L3 at 23°C followed by 26°C to L4. Levels of indicated mRNA from the cri-3_clpf-1 were quantified by RT-qPCR and normalized to act-1 mRNA levels with values of xrn-2(+) animals defined as 1 (n = 3, means ± SEM). Values are shown in S1 Table. (PDF) [file pgen.1006313.s004.pdf]

S5 Figure

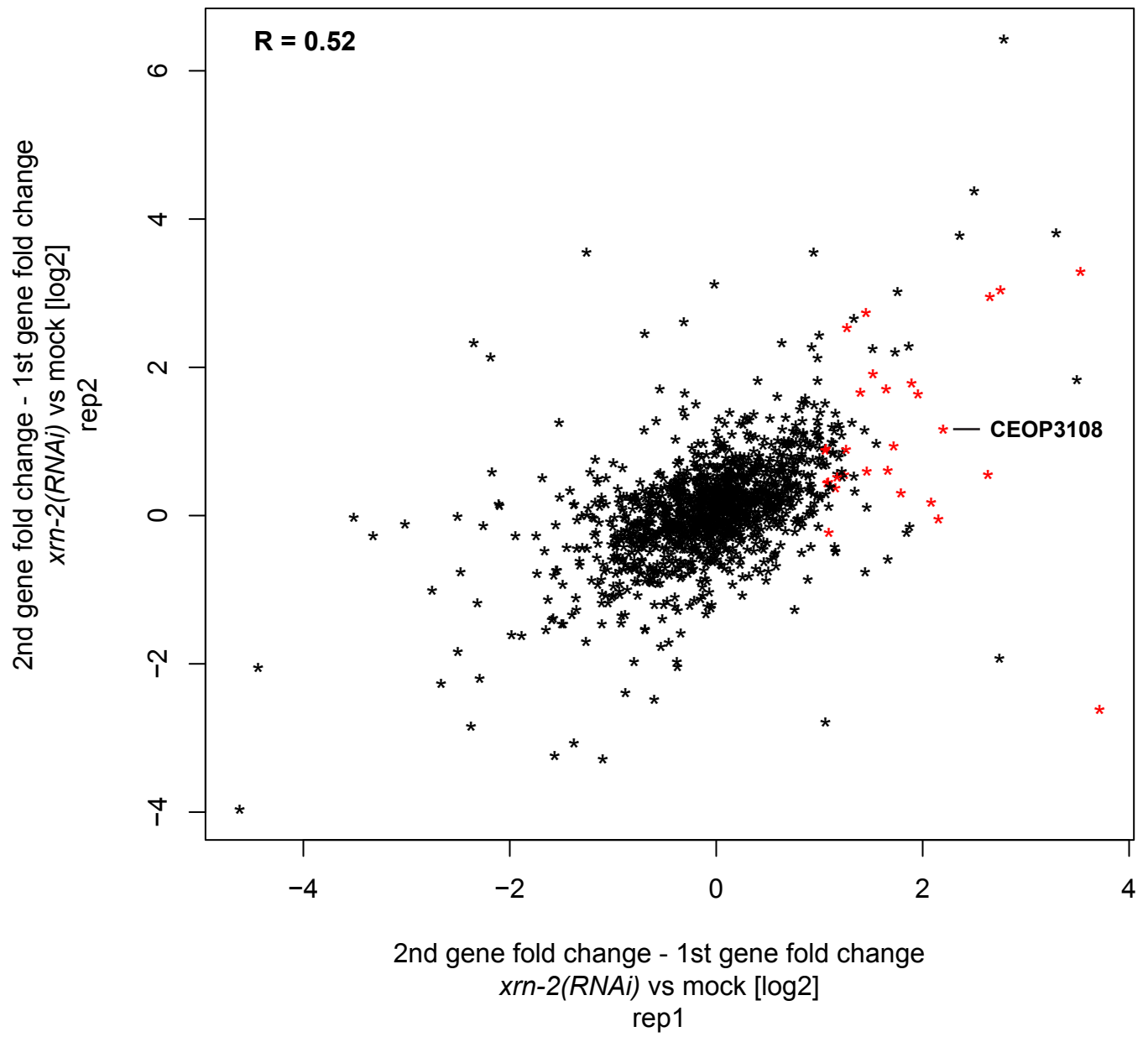

Supplement: S5 Fig — See Fig 7A and Materials and Methods. The difference in log2 fold-change between the second gene and the first gene in operons is plotted for each replicate against the other. Operons above the cut-off in replicate 1 are shown in red. The Pearson correlation coefficient between the fold-change differences for the two replicates is shown in the upper left. (PDF) [file pgen.1006313.s005.pdf]
