# Supplementary material for: XRN2 Autoregulation and Control of Polycistronic Gene Expresssion in Caenorhabditis elegans
Source: PLoS Genet. 2016 Sep 15;12(9):e1006313. doi: 10.1371/journal.pgen.1006313 (PMC5025045; doi:10.1371/journal.pgen.1006313)

S2 Figure

A

```
Sc Hal2p      1 MALERELLVATQAVRKASLLTKRIQ-------------SEVISHKDSTTI 37
Rn Bpnt1      1 MA------------SSHNVLMRLVASAYSIAQKAGTIVRCVIAEGDLGIV 38
Ce BPNT-1     1 MF------------NKASFLTRLVASSVRVSEAAGGLIKNVMAGGDLKII 38

Sc Hal2p     38 TK----NDNSPVTTGDYAAQTIIINAIKSNFPDDKVVGEESSSGLSDA-- 81
Rn Bpnt1     39 QKT---SATDLQTKADRMVQMSICSSLSRKFPKLTIIGEEDLPPGEVDQE 85
Ce BPNT-1    39 DKSEHGSGYDPQTEADRRAQYCIVQSLQKHFKNINIIGEEEDTTACPE-- 86

Sc Hal2p     82 -FVSGILNEIKANDEVYNKNYKKDDFLFTNDQFPLKSLEDVRQIIDFGNY 130
Rn Bpnt1     86 LIEDGQSEEIL--KQPCPSQY--------------------------SA- 106
Ce BPNT-1    87 -IEMGFSADVLQMERLMSTEL--------------------------KN- 108

Sc Hal2p    131 EGGRKGRFWCLDPIDGTKGFLRGE---------QFAVCLALIVDGVVQLG 171
Rn Bpnt1    107 -IKEEDLVVWVDPVDGTKEYT-------EGLLDNVTVLIGIAYEGKAIAG 148
Ce BPNT-1   109 -IQENDVVVWVDPLDGTSEVALAVKNKNMALLEQVTVLIGIAYKGRPVAG 157

Sc Hal2p    172 CIGCPNLVLSSYGAQDLKGHESFGYIFRAVRGLGAFYSPSSDAESWTKIH 221
Rn Bpnt1    149 IINQP------YYNYQAGPDAVLGRTIWGVLGLGAFGFQLKEAPAGKHII 192
Ce BPNT-1   158 IIHQP------YHE-------KLGRTVWAIQGCGVHGVVPATGNAQ-KIV 193

Sc Hal2p    222 VRHLKDTKDMITLEGVEKGHSSHDEQTAIKNKLNISKSLHLDSQAKY--C 269
Rn Bpnt1    193 TTTRSHSNKL-----------VTDCIAAMN-----PDNVLRVGGAGNKII 226
Ce BPNT-1   194 VTTRSHLSES-----------VSNALEALK-TRNLADSVEKVGGAGFKVL 231

Sc Hal2p    270 LLALGLADVYLRLPIKLSYQEKIWDHAAGNVIVHEAGGIHTDAMEDVPLD 319
Rn Bpnt1    227 QLIEGKASAY----VFASPGCKKWDTCAPEVILHAVGGKLTDIH-GNPLQ 271
Ce BPNT-1   232 KVLEGC-AAY----VFASAGCKKWDTCAVEAVLTAAGGTLTDIS-GRDIR 275

Sc Hal2p    320 FGNGRTLA-TKGVIASSG-----------PRELHDLVVSTSCDVIQSRNA 357
Rn Bpnt1    272 YDKEVKHMNSAGVLAALRN--YEYYASRVPESVKSALIP----------- 308
Ce BPNT-1   276 YEPGVQLNNTGGVLATASWVKHKDYIDTIPQEIKNMLPEISSKK------ 319
```

bpnt-1(xe22) (W → Stop)

B

Rattus norvegicus Bpnt1

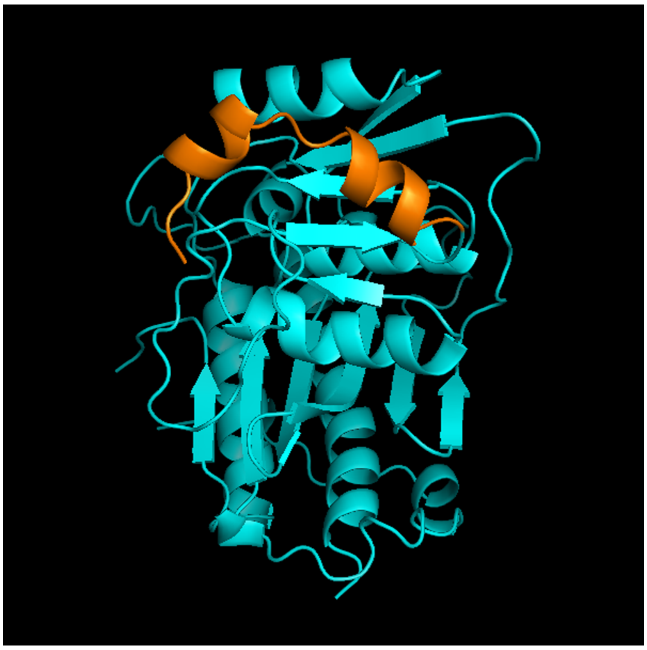

Supplement: S2 Fig — (A) Protein sequences of BPNT homologues in S. cerevisiae (Sc Hal2p), R. norvegicus (Rn Bpnt1) and C. elegans (Ce BPNT-1) are aligned by Clustal Omega (http://www.ebi.ac.uk/Tools/msa/clustalo/) and displayed by Jalview (http://www.jalview.org/).The most frequent amino acids are in blue, similar amino acids in light blue, based on BLOSUM62. Amino-acids numbers relative to the first methionine are shown. The bpnt-1(xe22) allele has a mutation that changes the 294th tryptophan to stop. (B) Structure of R. norvegicus Bpnt1. The corresponding region missing in the C. elegans BPNT-1(xe22/W294*) is shown in orange. (PDF) [file pgen.1006313.s002.pdf]
