# Supplementary material for: XRN2 Autoregulation and Control of Polycistronic Gene Expresssion in Caenorhabditis elegans
Source: PLoS Genet. 2016 Sep 15;12(9):e1006313. doi: 10.1371/journal.pgen.1006313 (PMC5025045; doi:10.1371/journal.pgen.1006313)

S6 Figure

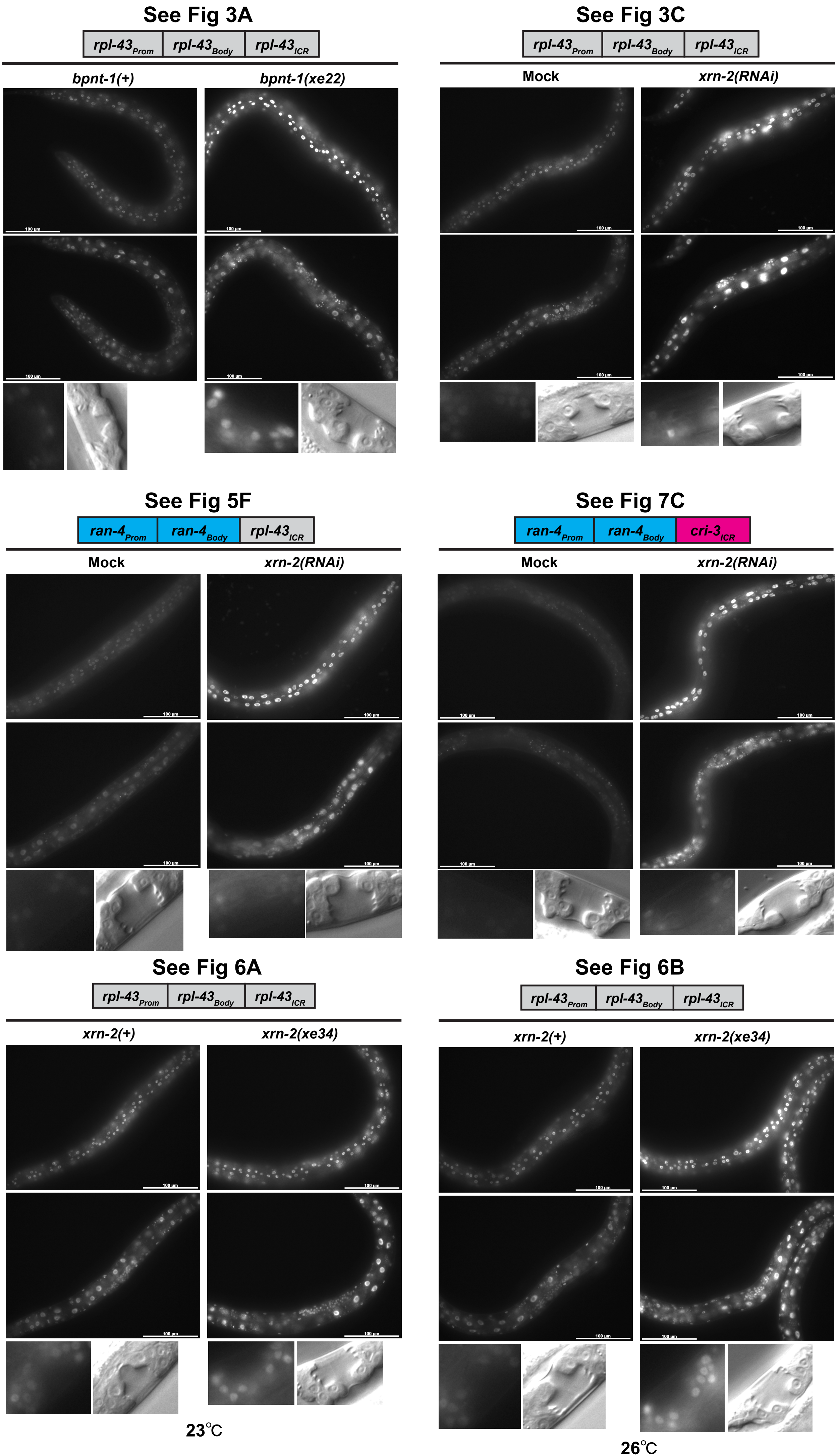
See Fig 3A
rpl-43Prom rpl-43Body rpl-43ICR
bpnt-1(+)
bpnt-1(xe22)
See Fig 3C
rpl-43Prom rpl-43Body rpl-43ICR
Mock
xrn-2(RNAi)
See Fig 5F
ran-4Prom ran-4Body rpl-43ICR
Mock
xrn-2(RNAi)
See Fig 7C
ran-4Prom ran-4Body cri-3ICR
Mock
xrn-2(RNAi)
See Fig 6A
rpl-43Prom rpl-43Body rpl-43ICR
xrn-2(+)
xrn-2(xe34)
23℃
See Fig 6B
rpl-43Prom rpl-43Body rpl-43ICR
xrn-2(+)
xrn-2(xe34)
26℃

See Fig 5G

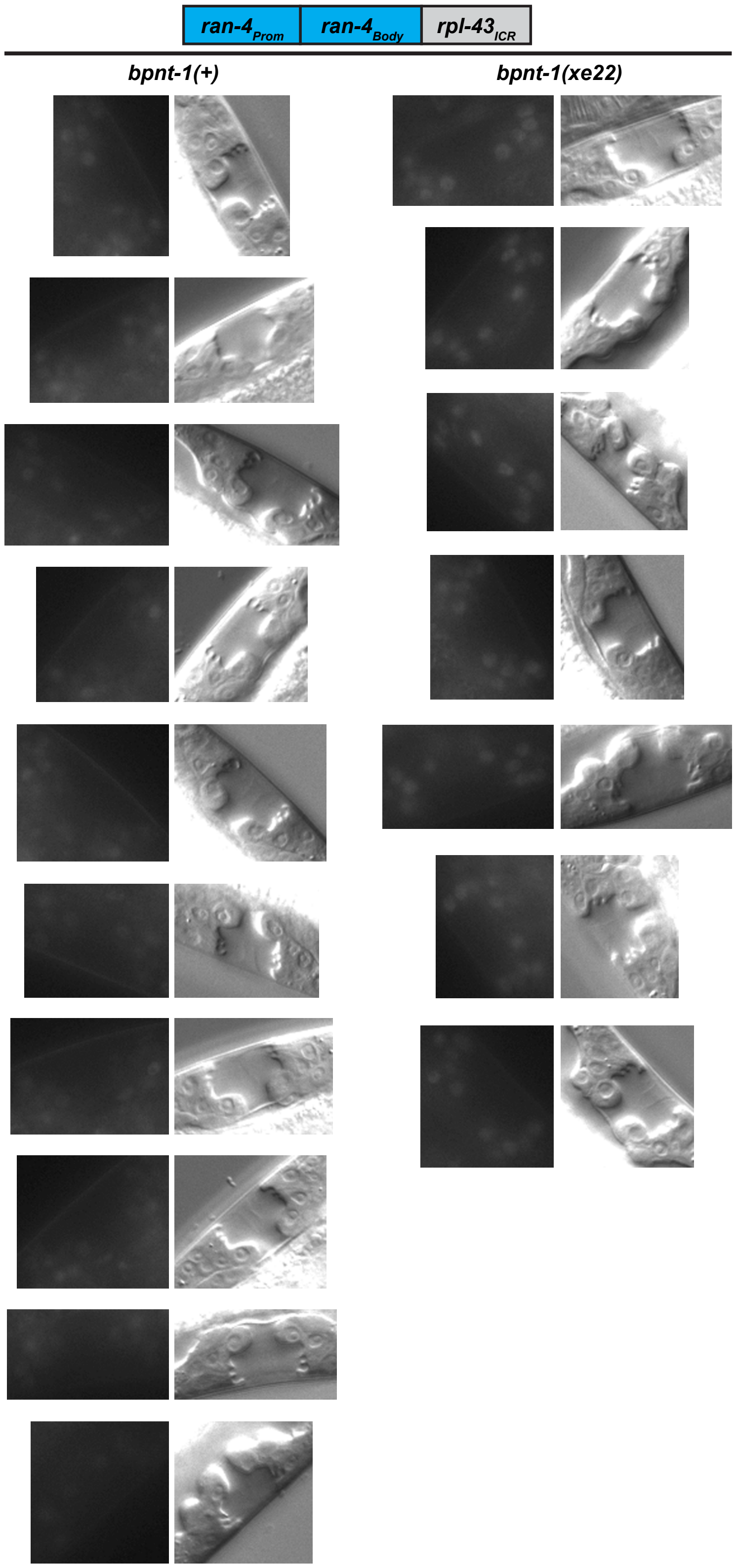

See Fig 7D

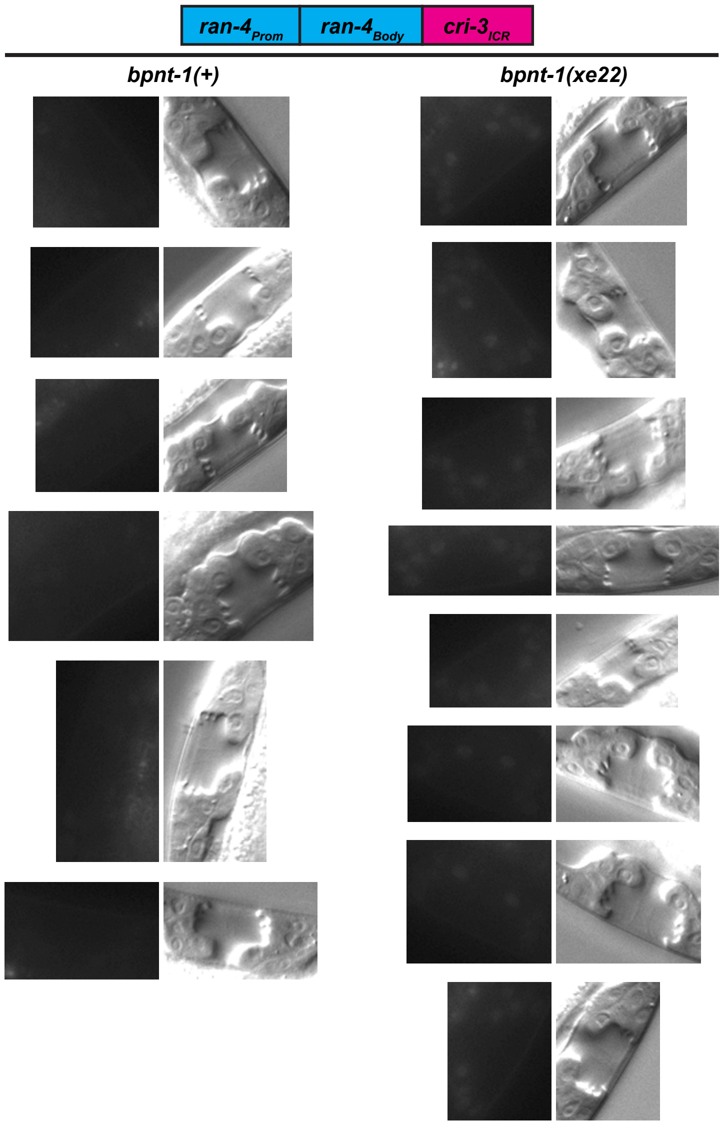

Supplement: S6 Fig — See Materials and Methods. (PDF) [file pgen.1006313.s006.pdf]
